# Evolutionary dynamics and molecular epidemiology of H1N1 pandemic 2009 influenza A viruses across swine farms in Denmark

**DOI:** 10.1093/ve/veaf014

**Published:** 2025-03-07

**Authors:** Sophie George, Pia Ryt-Hansen, Anders Gorm Pedersen, Klara M Anker, Jakob N Nissen, Jesper S Krog, Charlotte K Hjulsager, Ramona Trebbien, Lars E Larsen

**Affiliations:** Department of Veterinary and Animal Sciences, University of Copenhagen, Frederiksberg C 1870, Denmark; Department of Veterinary and Animal Sciences, University of Copenhagen, Frederiksberg C 1870, Denmark; Department of Health Technology, Section for Bioinformatics, Technical University of Denmark, Kgs. Lyngby, Hovedstaden 2800, Denmark; Department of Health Technology, Section for Bioinformatics, Technical University of Denmark, Kgs. Lyngby, Hovedstaden 2800, Denmark; Department of Virus and Microbiological Special Diagnostics, Statens Serum Institut, Copenhagen S, Hovedstaden 2300, Denmark; Department of Virus and Microbiological Special Diagnostics, Statens Serum Institut, Copenhagen S, Hovedstaden 2300, Denmark; Department of Virus and Microbiological Special Diagnostics, Statens Serum Institut, Copenhagen S, Hovedstaden 2300, Denmark; Department of Virus and Microbiological Special Diagnostics, Statens Serum Institut, Copenhagen S, Hovedstaden 2300, Denmark; Department of Virus and Microbiological Special Diagnostics, Statens Serum Institut, Copenhagen S, Hovedstaden 2300, Denmark; Department of Veterinary and Animal Sciences, University of Copenhagen, Frederiksberg C 1870, Denmark

**Keywords:** influenza A virus, H1N1 pandemic 2009, viral evolution, zoonosis, swine, human

## Abstract

Transmission of influenza A viruses (IAVs) between pigs and humans can trigger pandemics but more often cease as isolated infections without further spread in the new host species population. In Denmark, a major pig-producing country, the first two detections of human infections with swine-like IAVs were reported in 2021. These zoonotic IAVs were reassortants of the H1N1 pandemic 2009 lineage (“H1N1pdm09,” H1 lineage 1A, clade 1A.3.3.2) introduced to swine farms in Denmark through humans approximately 11 years prior. However, predicting the likelihood and outcome of such IAV spillovers is challenging without a better understanding of the viral determinants. This study traced the evolution of H1N1pdm09 from 207 sequenced genomes as the virus propagated across Danish swine farms over a decade. H1N1pdm09 diverged into several genetically distinct viral populations, largely prompted by reassortments with neuraminidase (NA) segments from other enzootic IAV lineages. The genomic segments encoding the viral envelope glycoproteins, hemagglutinin (HA) and NA, evolved at the fastest rates, while the M and NS genomic segments were among the lowest evolutionary rates. The two zoonotic IAVs emerged from separate viral populations and shared the highest number of amino acid mutations in the PB2 and HA proteins. Acquisition of additional predicted glycosylation sites on the HA proteins of the zoonotic IAVs may have facilitated infection of the human patients. Ultimately, the analysis provides a foundation from which to further explore viral genetic indicators of host adaptation and zoonotic risk.

## 1. Introduction

Influenza A viruses (IAVs) continuously infect human and farmed pig populations. Human inhabitants of temperate regions experience annual seasonal waves of IAV epidemics, while humans in the tropics and pig farms worldwide experience IAV respiratory infections throughout the year (Tamerius et al. 2013, Henritzi et al. 2020, Yin and Robertson 2021, Servadio et al. 2023). Maintaining circulation in a host population creates selective pressure for IAVs to evade host immune defenses and adapt to the host species (Long et al. 2019, Han et al. 2023). IAVs replicate eight individual genomic segments with a self-encoded, error-prone polymerase complex and package the replicated segments into new progeny virions (Boivin et al. 2010, Arranz et al. 2012). Consequently, evolution proceeds mainly through two mechanisms: the steady accumulation of replication nucleotide mutations, termed “genetic drift,” and the spontaneous reassortment of entire genomic segments between coinfecting IAVs, termed “genetic shift” (Michelle and Holmes 2020, Ganti et al. 2022). Selective and stochastic forces then act to propagate or purify the genetic variants over successive generations within and between hosts, leading to genetic diversification and host species-specific lineages of IAVs (Spielman et al. 2019, Amato et al. 2022). The classification of IAVs into numbered subtypes is based on the antigenicity of the viral envelope glycoproteins, hemagglutinin (HA) and neuraminidase (NA), and the genotype additionally considers the lineage of the other six “internal” genomic segments (Krammer et al. 2018).

Most spillovers of swine-specific IAVs (swIAVs) into humans, and human-specific (human seasonal) IAVs into swine, cause “dead-end” infections but carry the potential to progress into sustained transmission chains and even widespread pandemics (Myers et al. 2007, Anderson et al. 2021, Markin et al. 2023). The last global human IAV pandemic declared in 2009 was initiated by the zoonosis of a novel H1N1 genotype (H1N1pdm09) generated by sequential reassortments of swIAVs infecting pig farms in the Americas (Garten et al. 2009, Mena et al. 2016). H1N1pdm09 quickly spread worldwide among humans, causing similar levels of morbidity and mortality as typical epidemics but disproportionately affecting younger adults more severely (Morens et al. 2010, Dawood et al. 2012). The previous human seasonal H1N1 virus was soon replaced and H1N1pdm09 (HA H1 clade classification 6B.1) has since cocirculated in the human population with another human seasonal IAV of H3N2 subtype (Pica et al. 2012). H1N1pdm09 also spilled over from humans into European pig populations (termed “reverse zoonosis”) and became an enzootic swIAV lineage (HA H1 lineage 1A, clade classification 1A.3.3.2) infecting swine farms in Denmark since 2010 alongside other enzootic swIAVs of subtypes H1N1, H1N2, and H3N2 (Watson et al. 2015). Almost 11 years later in 2021, the first two separate cases of human infections with nonhuman seasonal IAVs were reported in Denmark (Nissen et al. 2021, Andersen et al. 2022). Both variant H1N1 (vH1N1) IAVs, A/Denmark/1/2021(vH1N1) and A/Denmark/36/2021(vH1N1), shared very high genetic similarity to contemporary reassortant swIAVs in Denmark and consisted mostly of gene segments from H1N1pdm09 swIAV lineage (1A.3.3.2) with one or two segments from H1N1 Eurasian avian-like swIAV lineage [H1N1av (HA H1 lineage 1C, clade classification 1C.2-like)].

Anticipating the emergence of such zoonotic swIAVs is difficult without a deeper comprehension of the viral adaptations required to infect and transmit between humans (Salvesen and Whitelaw 2021). Some progress has been made toward characterizing specific amino acids in the IAV proteins that enhance the viral fitness of H1N1pdm09 in the human host environment (Griffin and Mark Tompkins 2023). Examination of the H1N1pdm09 descendants in Danish swine farms offers further opportunities to discover more viral genetic markers of host adaptation and zoonotic potential. The intensive pig production in Denmark has been monitored by a nationwide, passive swIAV surveillance program established in the aftermath of the 2009 IAV pandemic and has amassed a large collection of swIAV samples from which to access the evolutionary history of H1N1pdm09 in pigs. Furthermore, the pig production industry in Denmark rarely imports live pigs, effectively creating a closed environment for H1N1pdm09 to evolve with limited interference of IAVs from other pig populations (Landbrug & Fødevarer n.d.).

A previous study examined a subset of swIAVs in Denmark between the years 2013 and 2018 and found a genetically diverse population of H1N1pdm09 and reassortants with NA segments from other circulating swIAV lineages (Ryt-Hansen et al. 2021). In this study, we sequenced the remaining H1N1pdm09 and H1pdm09-NA reassortant genomes (collectively termed “H1pdm09Nx”) from the Danish swIAV surveillance archives (collected from 2010 to 2020) and examined the evolution of H1pdm09Nx across Danish swine farms that lead to the emergence of the zoonotic IAVs. Available bioinformatics tools were applied to infer phylogenetic relationships, approximate genetic divergence events, estimate evolutionary rates, detect selection pressures on gene evolution, map the inheritance of amino acid mutations, and predict glycosylation of the HA protein.

## 2. Materials and methods

### 2.1. Virus sample collection

H1pdm09Nx samples (identified as containing the HA genomic segment of H1N1pdm09 origin) were retrieved from the archive of the Danish swIAV surveillance program. The samples included nasal swabs (∼60%), lung tissues (∼35%), and oral fluids (∼5%) collected from farmed pigs in Denmark upon clinical suspicion of IAV infection and submitted to the Danish swIAV surveillance program for clinical diagnostics between June 2010 and September 2020. Clinical diagnostics involved screening submissions for IAV, subtyping one IAV-positive sample per submission by reverse transcriptase quantitative polymerase chain reaction (RT-qPCR), and sequencing a selection of subtyped samples from each year as previously described (Ryt-Hansen et al. 2021). Primary samples were stored at −80°C until retrieval for the present study.

### 2.2. Viral RNA isolation

Archived nasal swab and oral fluid samples were centrifuged at 8000 g for 5 min and 200 µl supernatant was lysed in 400 µl RNeasy lysis buffer (QIAGEN) supplemented with 1% β-mercaptoethanol (Sigma-Aldrich). Archived lung tissue samples weighing 70 mg were lysed in 1.4 ml RNeasy lysis buffer with 1% β-mercaptoethanol, followed by homogenization at 30 Hz for 3 min using TissueLyser II (QIAGEN). Homogenates were centrifuged at 8000 g for 5 min to collect 600 µl supernatant. Total RNA extraction from lysed nasal swab, oral fluid, and lung tissue samples was automated using the QIAcube Connect robot (QIAGEN) with the RNeasy Mini kit (QIAGEN) according to the manufacturer’s instructions.

### 2.3. Whole-genome amplification

Viral RNA recovery was measured by RT-qPCR that targeted the matrix (M) genomic segment as previously described (Ryt-Hansen et al. 2021). Samples containing viral loads with a cycle threshold value of ≤32 proceeded to whole-genome amplification. All eight genomic segments were reverse transcribed and amplified from isolated RNA using the one-tube RT-PCR method with SuperScript™ III One-Step RT-PCR Platinum™ Taq HiFi (Invitrogen) and MBTuni-12(R)/MBTuni-13 primer pair as previously described (Zhou et al. 2009). PCR products were visualized on an e-gel (Invitrogen) to confirm amplification of IAV genomic segments and subsequently purified using the High Pure PCR Product Purification Kit (Roche).

### 2.4. Whole-genome sequencing

Whole-genome sequencing libraries were constructed from 0.2 ng/µl of diluted whole-genome amplified PCR products using the Nextera XT DNA library preparation kit (Illumina). Libraries were normalized to 2 nM and pooled before sequencing on the MiSeq Illumina platform using the MiSeq V2 500 cycles kit according to the manufacturer’s instructions. Previously sequenced samples from the Danish swIAV surveillance collection were resequenced as repeat controls (*n* = 20).

### 2.5. Genome sequence assembly

All steps involved in genome sequence reconstruction were performed in CLC Genomics Workbench versions 20.0.4 to 22.0.2 (QIAGEN). MiSeq sequencing reads were paired and trimmed under default quality controls (remove failed reads, quality scores from National Center for Biotechnology Information (NCBI)/Sanger or Illumina Pipeline 1.8 later, trim using quality scores 0.05, and automatic read-through adapter trimming). A panel of reference IAV segment sequences, representing the lineages circulating in swine (H1N1pdm09, H1N1av, H3N2 Hong Kong, and H1N2UK), guided read assembly into genomic segment sequences. Sequence reads assembling to multiple genomic segment reference lineages were discarded to limit unreliable genome reconstruction with coinfected samples (further verification is described in Section 2.10). Consensus full-length segment sequences with a minimum coverage depth of 100× were extracted for each genomic segment from every sample. Segments without 100× coverage across the entire length were discarded and reconstructed genomes with missing segments were referred to as “partial” genomes.

### 2.6. Additional sequencing data collection

All Danish swIAV H1pdm09Nx sequences available in nucleotide sequence databases, GISAID EpiFlu™ (https://www.gisaid.org/, last accessed January 2022) and NCBI GenBank (https://www.ncbi.nlm.nih.gov/genbank/, last accessed January 2022) and the zoonotic IAVs detected in Denmark (A/Denmark/1/2021(vH1N1) and A/Denmark/36/2021(vH1N1)) were incorporated into the Danish swIAV H1pdm09Nx sequence dataset. Every segment sequence was entered into BLAST searches in the sequence databases to download genetically related IAVs isolated from non-Danish swine farms (*n* = 141), humans [*n* = 103, including World Health Organization–recommended vaccine strains (A/California/07/2009, A/Michigan/45/2015, A/Brisbane/02/2018, and A/Guangdong-Maonan/SWL1536/2019; World Health Organization n.d.)], and avian species (*n* = 2). A list of all IAVs included in the analyses is provided in the Supplementary material (isolates.xlsx) and the accession numbers for Danish swIAV H1pdm09Nx sequences are listed in Supplementary Table 1.

### 2.7. Subtype-lineage classification

Danish swIAV H1pdm09Nx genomes were initially classified into subtypes and lineages according to preferential mapping to reference IAV segments during genome assembly, then later confirmed by phylogenetic analysis. HA genes of H1pdm09 origin (clade 1A.3.3.2) were additionally verified using Swine H1 Clade Classification Tool (Anderson et al. 2016). NA lineages were defined according to their origin; “N1pdm09” from H1N1pdm09, “N1av” from 1970s H1N1av, “N2sw” from A/swine/Gent/84 H3N2-like, and “N2hu” from 1990s human seasonal H3N2-like IAVs, as previously classified (Ryt-Hansen et al. 2021). H1pdm09Nx genomes containing segments from non-H1N1pdm09 origin were recognized as reassortant IAVs.

### 2.8. Phylogenetic molecular clock and evolutionary rate analysis

Consensus sequences from Danish swIAV H1pdm09Nx and related IAVs downloaded from the sequence databases were aligned per genomic segment using MAFFT v7.515 and noncoding regions were removed (Kazutaka and Standley 2013). The best fitting substitution models for phylogenetic analyses were determined for each segment sequence alignment by ModelFinder (Subha et al. 2017). Maximum likelihood phylogenetic trees were reconstructed from each segment sequence alignment by IQ-TREE v2.0.3 using the selected substitution models with ultrafast bootstrap approximation set to 1000 and maximum number of iterations limited to 2500 (Minh et al. 2020). An example of the command used to run the model is *iqtree –safe –s alignment.fa –nt 2 –m GTR+F+I+G4 –nm 2500 –bb 1000*. Output files from IQ-TREE analysis are available in the Supplementary material (prefixed with “iqtree_”). Consensus maximum likelihood phylogenetic trees were assessed for temporal signal using the software TempEST v1.5.3 with the best-fitting root function (Rambaut et al. 2016). Root-to-tip regression correlation coefficients (*r*^2^) measuring >0.8 indicated suitability for molecular clock analysis and estimation of evolutionary rates. The consensus maximum likelihood phylogenetic trees, segment sequence alignments, and IAV sampling dates were used as input to the software TreeTime v0.9.2, which reconstructed phylogenetic molecular clock trees with a strict molecular clock model and an allowance for covariation of evolutionary rates across the phylogeny (Sagulenko et al. 2018). An example of the command used to run the model is *treetime –tree iq-tree.nwk –aln alignment.nxs –dates dates.csv –reconstruct-tip-states –covariation –confidence*. Time scales were fitted onto molecular clock phylogenetic trees in FigTree v1.4.4 software (http://tree.bio.ed.ac.uk/software/Figtree/) by reversing the scale axis and offsetting the time scale to the numerical collection date of the most recent isolate.

### 2.9. Phylogenetic clade determination and genetic divergence estimation

For each segment phylogenetic tree, preliminary clades were appointed to clusters of sequences that descended from single common ancestors and shared common genetic characteristics. Then finalized clade designations were given to the preliminary clades that formed across all eight segment phylogenies and consisted of mostly the same IAVs with a commonly identified IAV close to the root of the clade. Clade determinations were further supported by higher average pairwise sequence identity among sequences within clades than average pairwise sequence identity between clades. Formulated clade nomenclature was based on common characteristics shared by the clustering isolates, such as reassortment with another NA gene lineage or common IAVs located close to the root of the clade.

Using the estimated sampling dates of the internal ancestral nodes in the phylogenetic trees, the date at which the clade began to genetically diverge was approximated by the date of the most recent common ancestor (MRCA) shared between the clade-forming branch and the remainder of the phylogenetic tree.

### 2.10. Selection detection

Selective pressures acting across the phylogenies at the codon level were assessed using HyPhy package and web application Datamonkey (Weaver et al. 2018; Pond et al. 2020). First, phylogenies were screened for the absence of genetic recombination using the genetic algorithm for recombination detection tool, which lent further assurance that genomes were assembled from single IAV infections as recombination in IAV evolution is extremely rare (Pond et al. 2006, Shao et al. 2017). Lineage-specific selection, whereby a proportion of sites along a branch were subjected to episodic evolution, was tested by an adaptive branch-site random effects likelihood model (aBSREL) with recommended significance set at *P*-value ≤ .05 (Smith et al. 2015). Test branches were selected on phylogenetic trees generated in Datamonkey by highlighting branch paths from the root to the deepest node in the defined phylogenetic clades. Paths leading from zoonotic and reverse zoonotic terminal branches were additionally selected as test branches.

To detect selection acting on evolution at the gene and individual codon level, coding sequences for each of the 10 proteins in the “core” IAV proteome, plus the accessory protein PA-X, were extracted from the segment sequences. Matrix 1 protein (M1) and matrix 2 protein (M2) genes overlap on the coding region of the M gene segment at nucleotide positions 1–27 and 716–759, and nonstructural 1 protein (NS1) and nuclear export protein (NEP) genes overlap on the coding region of the NS segment (H1N1pdm09 lineage) at 1–23 and 496–660. PA-X, encoded by segment 3 and translated by a +1 ribosomal frameshift at 573 nucleotides into the polymerase acidic protein (PA) gene, was included due to contribution toward the shutoff of host gene expression during IAV infection in humans (Hayashi et al. 2016). Presence of pervasive positive and negative purifying selection on the evolution of each gene was detected using fixed effects likelihood (FEL) and single-likelihood ancestor counting (SLAC) methods with recommended significance thresholds set at *P*-value ≤ .1 (Pond and Sergei 2005). Episodic positive selection of codon sites was detected using mixed effects model of evolution (MEME) with recommended significance threshold set at *P*-value ≤ .1 (Murrell et al. 2012).

### 2.11. Ancestral reconstruction and amino acid mutation inheritance

Hypothetical sequences of the phylogenetic ancestral nodes were predicted using ancestral reconstruction features in both TreeTime v0.9.2, for tree formatting, and CodeML, for data handling purposes (Yang 1997). Amino acid substitutions were extracted from the branches stemming from the first ancestral node to the MRCA of each defined phylogenetic clade and from all the internal branches within the clade, such that a substitution was inherited by at least two IAVs but not necessarily by all IAVs in the clade. Amino acid substitutions inherited by the zoonotic IAVs, A/Denmark/1/2021(vH1N1) and A/Denmark/36/2021(vH1N1), were extracted separately and compared to identify commonly mutated protein sites.

### 2.12. Prediction of post-translational glycosylation modifications to HA protein

N-linked glycosylation sites were predicted on HA using a neural network-based model, NetNGlyc 1.0, hosted at https://services.healthtech.dtu.dk/ (Gupta and Brunak 2002, Steentoft et al. 2013). Basic N-linked glycan structures were attached to the predicted N-linked glycosylation sites on a structural homotrimer model of HA from A/Denmark/36/2021(vH1N1) (modeled by SWISS-MODEL-automated protein structure homology-modeling server; Waterhouse et al. 2018) using the Glyprot webserver and optimized by energy minimization using the YASARA minimization server (Bohne-Lang and von der Lieth 2005, Land and Svedendahl Humble 2018). The resulting glycosylated model was formatted in PyMOL software (Schrödinger).

## 3. Results

### 3.1. H1pdm09Nx genome sequence recovery

**Figure 1.**
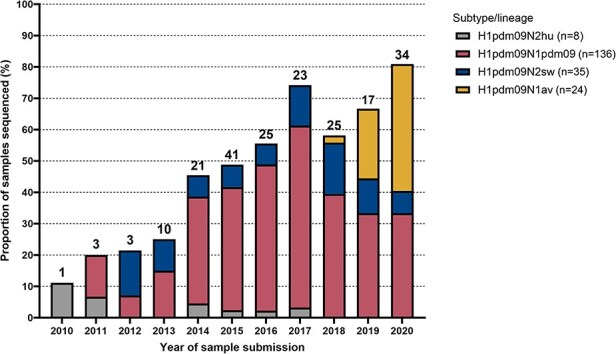
Proportion of H1 pandemic 2009 (H1pdm09Nx) genomes sequenced from the Danish swine influenza A virus surveillance collection per year. Distribution of HA–NA subtypes and lineages (defined in Methods) is indicated and number of samples sequenced are provided above the corresponding bar.

Retrospective sequencing of samples collected from pigs farmed in Denmark between June 2010 and September 2020 recovered 139 full (eight segments) and partial (two to seven segments) H1pdm09Nx genomes. With the supplementation of all previously sequenced H1pdm09Nx samples (*n* = 64) collected during 2011–19, the sequence dataset totaled 203 Danish swIAV H1pdm09Nx genomes (176 full and 27 partial genomes) and represented 11–81% of annual H1pdm09Nx submissions received by the Danish swIAV surveillance program (Fig. 1).

Scant coverage of the earlier years (2010–15) largely missed the initial genetic diversity of the H1N1pdm09 spillovers from humans. Nevertheless, the wider range of submissions sequenced in the later years (2016–20) likely captured the major prevailing genetic lineages, and the subtype-lineage distribution of the sequenced genomes closely resembled the trends recorded in the annual Danish swIAV surveillance program reports, such as the absence of H1pdm09N2hu detections after 2017 and increased prevalence of H1pdm09N1av since 2018 (Ryt-Hansen et al. 2022). Therefore, the recovered genomes likely provided an adequate representation of the H1pdm09Nx genetic diversity in Danish swine farms between 2010 and 2020.

The majority of sequenced H1pdm09Nx genomes retained the NA genomic segment from the H1N1pdm09 lineage (67%) and the remainder incorporated NA genomic segments from reassortments with N2sw (17%), N1av (12%), and N2hu (4%) swIAVs. Reassortments of H1pdm09Nx involving other genomic segments were rarely detected but included five H1pdm09Nx genomes with an NS genomic segment of H1N1av origin and one H1N1pdm09 genome with internal genomic segments derived from other enzootic swIAV lineages.

In addition, available sequences from four swIAV H1pdm09Nx samples collected in 2021 were included in subsequent analyses due to close genetic similarity to A/Denmark/36/2021(vH1N1) (A/Denmark/1/2021(vH1N1) was most closely related to swIAVs sampled in April 2020, which were already present in the sequence dataset) (Nissen et al. 2021, Andersen et al. 2022).

### 3.2. Genetic divergence of H1pdm09Nx in Danish swine farms

Phylogenetic molecular clock analyses reconstructed the evolution of H1pdm09Nx genomes in Danish swine farms. The resulting time-scaled phylogenetic trees, inferred separately for each genomic segment, revealed the divergence of multiple distinct H1pdm09Nx populations (Fig. 2a–i). IAV genomes in these populations largely exhibited consistent clustering tendencies across the segment phylogenetic trees, while IAV genomes with inconsistent clustering patterns inherited segments from multiple H1pdm09Nx ancestors (a list of Danish swIAV H1pdm09Nx and corresponding clade designations are presented in Supplementary Table 1).

**Figure 2.**
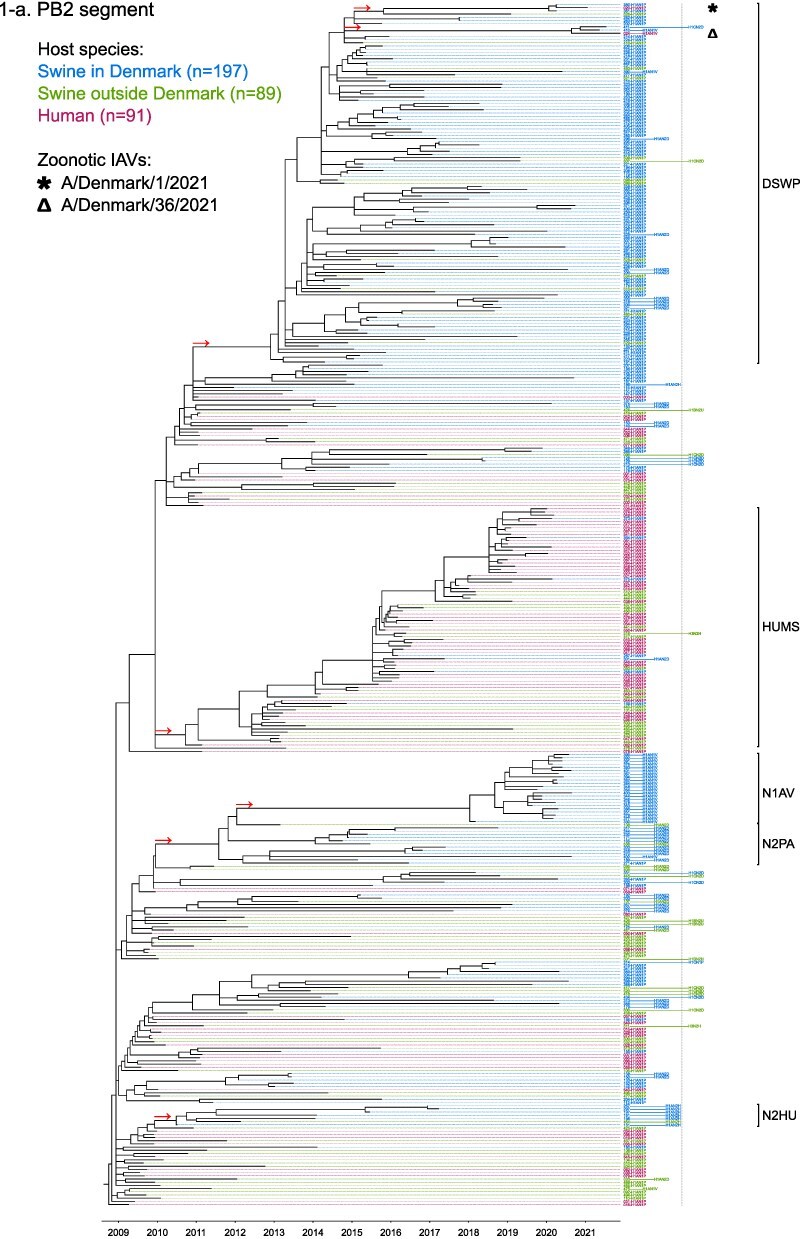
(a-l) Time-scaled phylogenetic trees of H1 pandemic 2009 (H1pdm09Nx) genomic segments. The genomic segment, expressed proteins (if segment encodes multiple proteins), and lineage origin (if non-H1pdm09Nx origin) are indicated. Branch length is represented by black lines and tip labels connected by dotted lines are colored according to the host species. Tip labels additionally correspond to HA–NA subtype and lineage of the sample and a gray dotted line separates H1pdm09Nx subtypes (left) from non-H1pdm09Nx subtypes (right). Zoonotic IAVs are marked by symbols and gray scale bars indicate amino acid (AA) lengths of expressed proteins PA-X and NS1 as presented in the phylogeny legend. Cross-segment clades were assigned to clusters of IAVs based on common characteristics and red arrows indicate the divergence of clades and zoonotic IAVs from the MRCAs.

**Figure 2.**
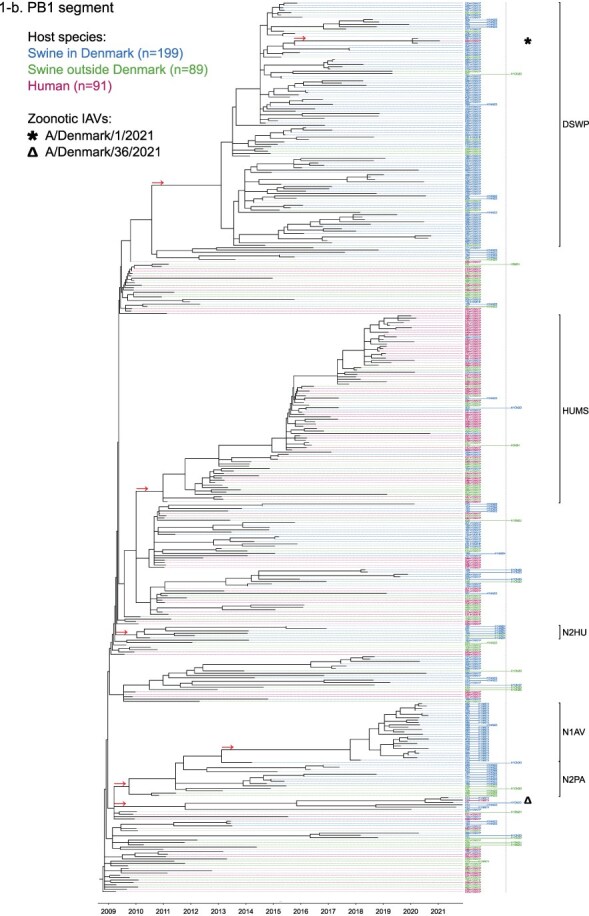
(Continued)

**Figure 2.**
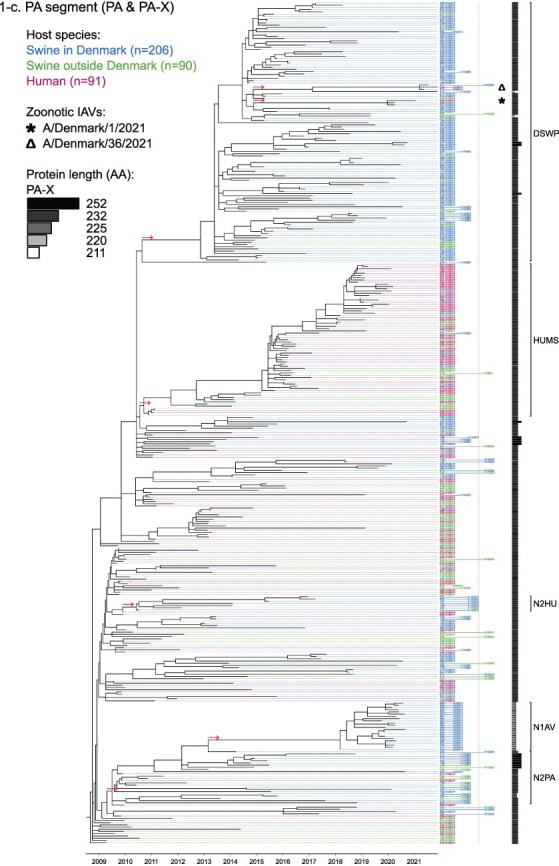
(Continued)

**Figure 2.**
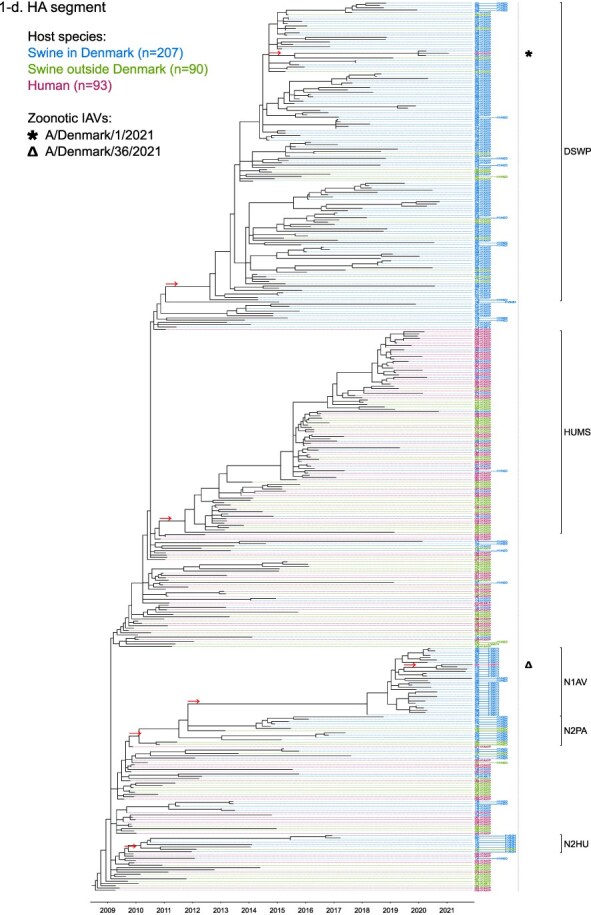
(Continued)

**Figure 2.**
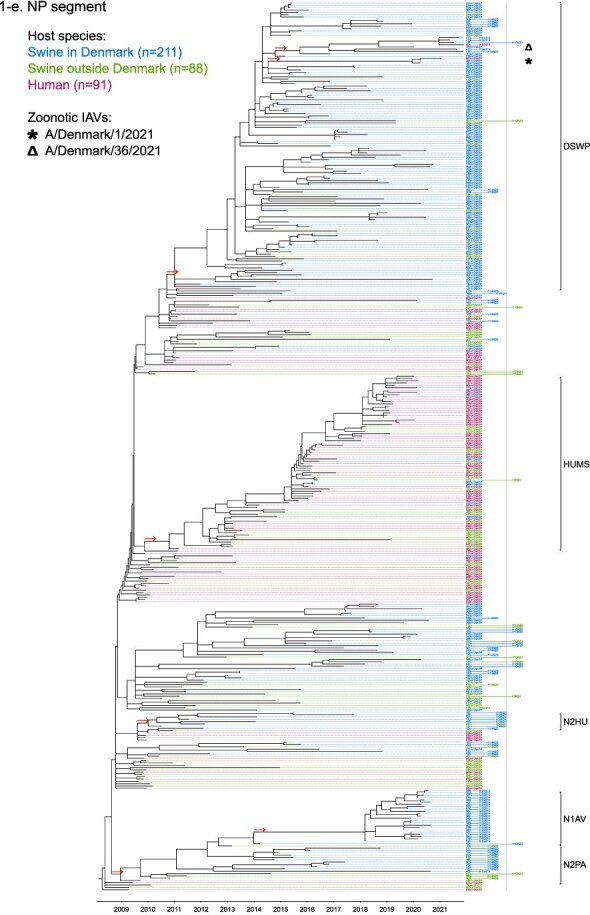
(Continued)

**Figure 2.**
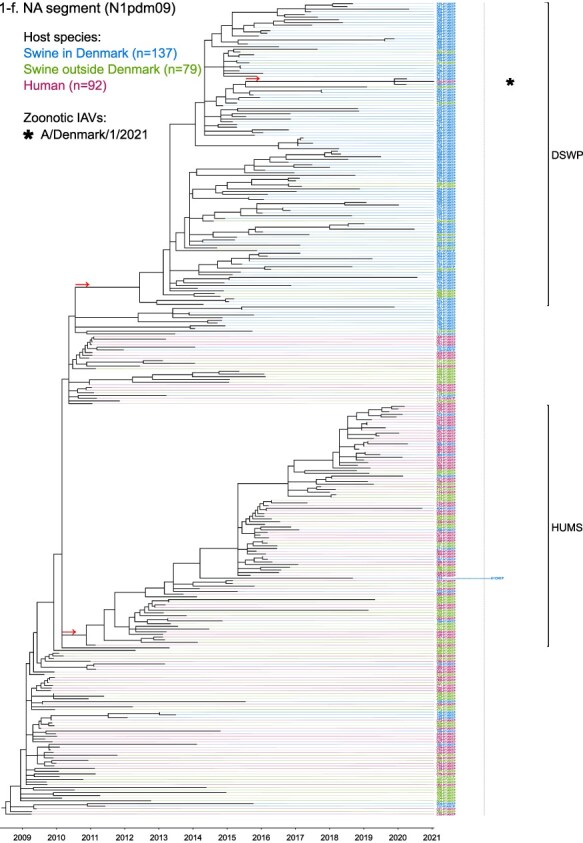
(Continued)

**Figure 2.**
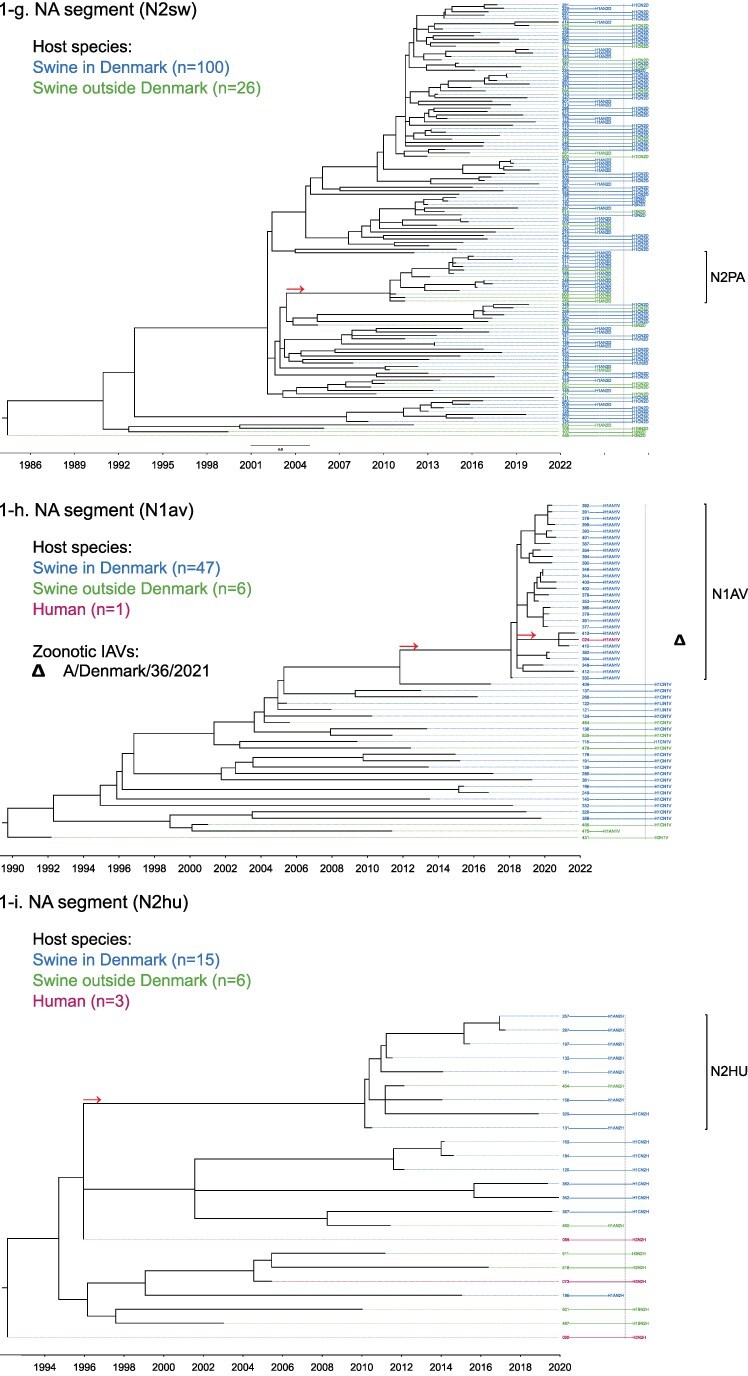
(Continued)

**Figure 2.**
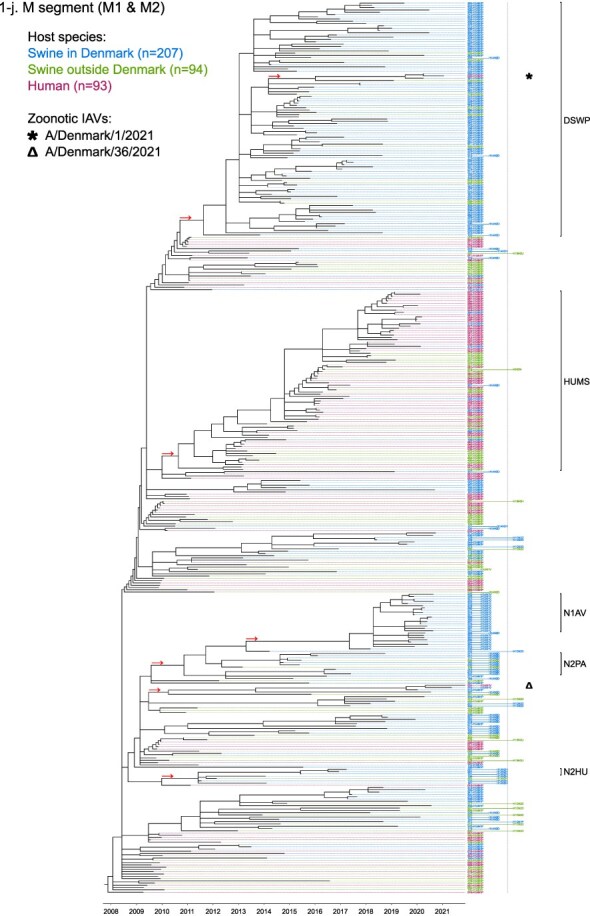
(Continued)

**Figure 2.**
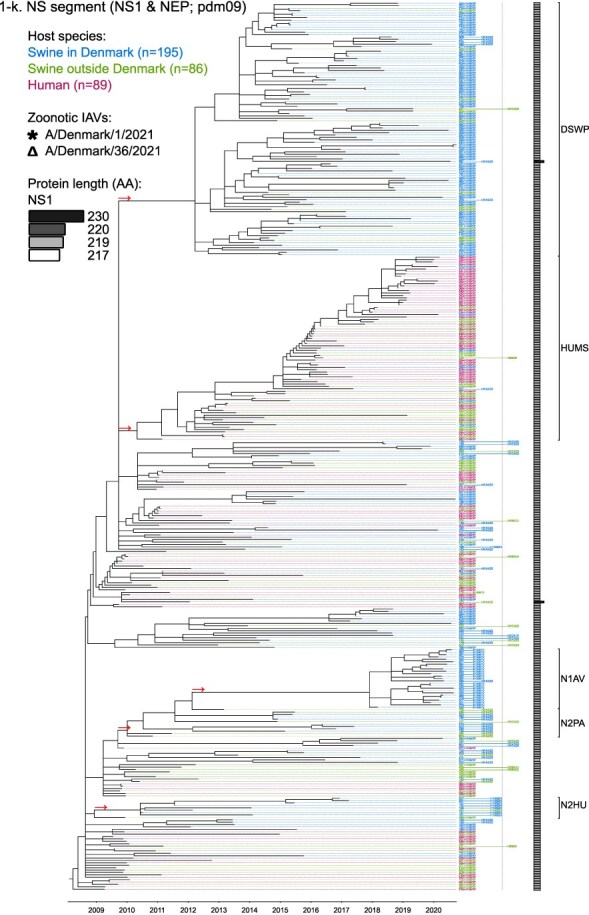
(Continued)

**Figure 2.**
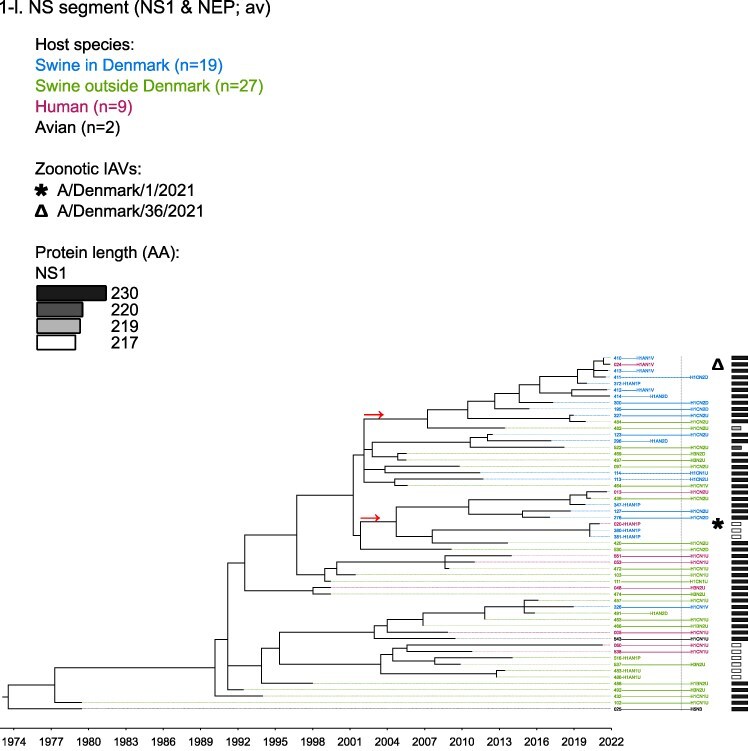
(Continued)

The majority of H1N1pdm09 full genomes (59%) consistently clustered into clade DSWP (abbreviation of “Danish swIAV H1N1pdm09”) and had descended from a single common ancestor. Using the approximated dates of the MRCAs shared between the clade-forming branch and the remainder of the phylogenetic tree as the indicator of clade initiation, clade DSWP began forming in late 2009 (NS segment) to early 2011 (HA segment). Thereafter, relatively long clade-forming branches extended 3–4 years before the rapid diversification and expansion into several offshoots of subclusters in clade DSWP.

H1pdm09N2sw reassortant genomes observed more erratic clustering across the segment phylogenetic trees. Multiple spillovers of H1N1pdm09 from humans during 2009–11 reassorted with a genetically diverse pool of NA segments from N2sw swIAVs that shared MRCAs in early 2002 (Fig. 2g). Although, almost a quarter of H1pdm09N2sw reassortant genomes (24%) consistently clustered together to form clade N2PA and had descended from a single common ancestor shared with an H1pdm09N2sw detected in Germany (A/swine/Papenburg/IDT12653/2010) (Zell et al. 2020). The genomic segments began diverging in late 2008 [nucleoprotein (NP) segment] to early 2010 (NS segment).

Almost all H1pdm09N1av reassortant genomes (92%) clustered into clade N1AV and descended from a single reassortment event between an ancestor of H1pdm09N2sw in clade N2PA and an NA segment of H1N1av lineage. The timing of the reassortment event is unclear but most likely occurred after late 2011 in accordance with the divergence of the NA gene from the ancestral H1N1av population (Fig. 2h). The first H1pdm09N1av was detected in early 2018 following an approximated 4–6 years divergence from clade N2PA. Descendants of the H1pdm09N1av reassortment proliferated over the following 2 years with 33–50% of the sequenced H1pdm09Nx submissions from 2019 to 2020 clustering into clade N1AV. An observation exclusive to clade N1AV is the additional C-terminal truncation to accessory protein PA-X, reducing the protein length to 220 amino acids (Fig. 2c).

The earliest H1pdm09Nx detected in Danish swine farms (June 2010) had descended from a reassortment of an H1N1pdm09 spillover with an NA segment of a previous human seasonal H3N2 spillover that occurred in late 1995 (Fig. 2i). All descendant H1pdm09N2hu reassortant genomes clustered together to form clade N2HU. The NA genes of Danish swIAV H1pdm09N2hu in clade N2HU uniquely expressed an additional 1–4 amino acids (E/K-K-E-K/R) inserted after codon site 73 (N2 numbering) (sequence alignments are available in the Supplementary material (prefixed with “alignment_”). The other segments in clade N2HU began to genetically diverge between late 2008 (NS segment) and mid-2011 (M segment). Detection of H1pdm09N2hu in clade N2HU had been infrequent with the last case reported in early 2017.

Several Danish swIAVs (*n* = 7–12 per segment phylogeny), sampled between 2013 and 2020, were most closely related to human seasonal H1N1pdm09 (H1 clade 6B.1) IAVs and clustered together to form clade HUMS (abbreviation of “human seasonal H1N1pdm09”). Detections of human seasonal H1N1pdm09-like IAVs in Danish swine farms indicated the reoccurrence of reverse zoonotic spillovers. However, onward pig-to-pig transmission of reverse zoonotic IAVs appeared restricted without further detections of descendants, and reassortments with other swIAV lineages seemed limited. Clade HUMS had begun diverging in late 2009 (NS segment) to late 2010 (HA segment).

The two zoonotic swIAVs (A/Denmark/1/2021(vH1N1) and A/Denmark/36/2021(vH1N1)) were independently contracted 11 months apart in 2021 and their genomic segments vary in genetic relatedness. Their most closely related segments, polymerase basic 2 protein (PB2), PA, and NP, clustered into clade DSWP and shared the last MRCA in late 2014 (PB2 segment). With the exception of the NS segment, all remaining segments of A/Denmark/1/2021(vH1N1) also descended from clade DSWP with MRCAs dated from early 2014 (M segment) to mid-2015 (HA segment). A/Denmark/36/2021(vH1N1) had HA and NA segments that clustered into clade N1AV, while the polymerase basic 1 protein (PB1) and M segments diverged from other H1N1pdm09 ancestors elsewhere in the phylogeny. Reassortment events leading to the incorporation of NS segments from H1N1av (NSav) into H1pdm09Nx genomes were rarely observed prior to 2020, yet the zoonotic cases emerged from two such reassortments with NSav segments that shared an MRCA in early 2001 (Fig. 2l). Probable timings of these two independent reassortment events ranged from mid-2007 to early 2020 for the A/Denmark/1/2021(vH1N1)-associated IAV population and mid-2014 to early 2016 for the A/Denmark/36/2021(vH1N1)-associated IAV population. In addition, A/Denmark/1/2021(vH1N1) expressed a C-terminally truncated NS1 protein measuring 217 amino acids, uncharacteristic for H1N1av that typically express NS1 as 230 amino acids, but similar to H1N1pdm09 that express NS1 as 219 amino acids (Fig. 2k–l).

In summary, all segments of defined H1pdm09Nx clades had begun to genetically differentiate by approximately early 2010 to late 2013 and evolved separately over the subsequent 7–10 years with clade DSWP and clade N1AV becoming the two major H1pdm09Nx swIAV clades by numbers of clustering IAVs. Reverse zoonosis of human seasonal H1N1pdm09 was detected on at least 12 occasions after the genetic divergence of H1pdm09Nx swIAVs and human seasonal H1N1pdm09, while the first two reported zoonotic IAVs in Denmark emerged from swIAVs circulating in Danish swine farms during 2020–21.

### 3.3. Evolution rates of H1pdm09Nx genomic segments

Considering the number of years each clade has been evolving along individual trajectories, the speed in which nucleotide mutations propagate in the genomic segments can allude to the amount of genetic diversity likely to accumulate between the H1pdm09Nx populations and the frequency of genetic changes required to maintain circulation. The evolutionary rate for each segment was estimated by the phylogenetic molecular clock analysis and averaged across the entire phylogeny under the presumption that rates can vary between branches.

**Figure 3.**
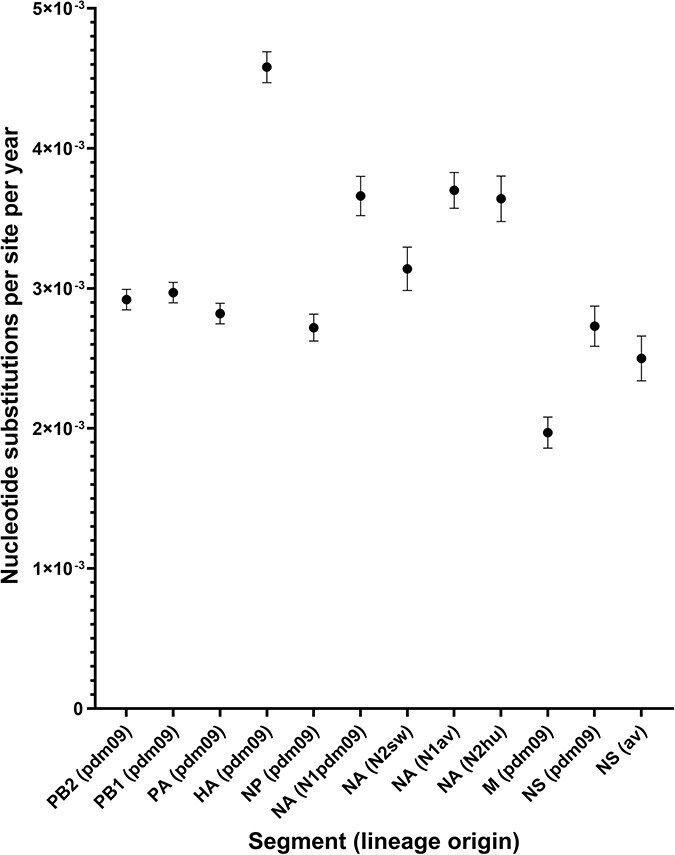
Rates of H1 pandemic 2009 (H1pdm09Nx) genomic segment evolution expressed as the number of nucleotide substitutions per site per year averaged across the genomic segment phylogeny. Rates were estimated by molecular clock analysis performed using TreeTime and plotted with one standard deviation of the mean rate. Supporting data are provided in Supplementary Table 2.

Segments encoding the viral envelope glycoproteins, HA (H1pdm09 lineage) and NA (N1pdm09, N1av, N2sw, and N2hu lineages), evolved at the fastest rate of 4.6 × 10^−3^ and 3.1 to 3.7 × 10^−3^ nucleotide substitutions per site per year (ns/s/y), respectively, while the slowest clock rate measured 2.0 × 10^−3^ ns/s/y for the M segment with overlapping genes that encode the M1 and M2 proteins, and the evolutionary rates of the remaining segments ranged between 2.5 and 3.0 × 10^−3^ ns/s/y (Fig. 3, Supplementary Table 2). These rates fall within the magnitude of typical IAV evolution estimates at ∼10^−3^ ns/s/y (Michelle and Holmes 2020). Statistical tests, *r*^2^ and *χ*^2^, indicate the level of variation between the expected genetic distance from the root in relation to the sampling date and the actual values of the sequences. *χ*^2^ scores were highest for HA and the three segments encoding the polymerase complex proteins (PB2, PB1, and PA), likely implying that a range of rates varied across the phylogeny, while the lowest scores were yielded by M and NS segments (excluding phylogenies with <100 samples), wherein nucleotide substitution rates were possibly more consistent across the phylogeny (Supplementary Table 2).

### 3.4. Selection pressures acting on H1pdm09Nx evolution

As positive selection can fix adaptive nucleotide mutations in a viral population or allow regressive nucleotide mutations to propagate when relaxed, the phylogenies were assessed for evolution under selective pressures using a series of selection detection models.

A branch-site method, aBSREL, was applied to test if positive episodic (or diversifying) selection impacted the emergence of the clades in each segment phylogeny. Positive selection, measured as nonsynonymous to synonymous codon substitution rate ratios (*dN*/*dS* or *ω*) > 1, was only detected on a proportion of codons evolving along two clade-forming branches in the HA segment phylogeny: one branch spanning 1.6 years into clade DSWP and another extending 6.3 years into clade N1AV (Supplementary Table 3). The model does not specify the codon sites that evolved under positive selection, but noticeably, 43% and 32% of the amino acid substitutions occurring along the two branches (clade DSWP and clade N1AV branches, respectively) fall within the antigenic sites on the HA protein.

Selection pressures acting on the evolution of the entire gene were assessed by the MEME and SLAC models on the 11 separated gene phylogenies encoding the 11 IAV proteins. All genes measured *ω* < 1, indicating negative purifying selection, as expected at the gene-wide level (Supplementary Table 4). The lowest *ω* ratios were calculated for M1 (0.05), NP (0.07–0.08 per model), PB2 (0.09–0.10 per model), PB1 (0.09–0.10 per model), and PA (0.10–0.11 per model) genes, possibly suggesting that the expressed proteins have stronger evolutionary constraints. The highest *ω* ratios were detected in M2 (0.55–0.58 per model), NS1 from both pdm09 (0.35–0.36 per model) and av lineages (0.31–0.33 per model), and HA (0.27–0.29 per model) genes, possibly due to higher evolutionary flexibility to respond to greater selection pressures.

**Figure 4.**
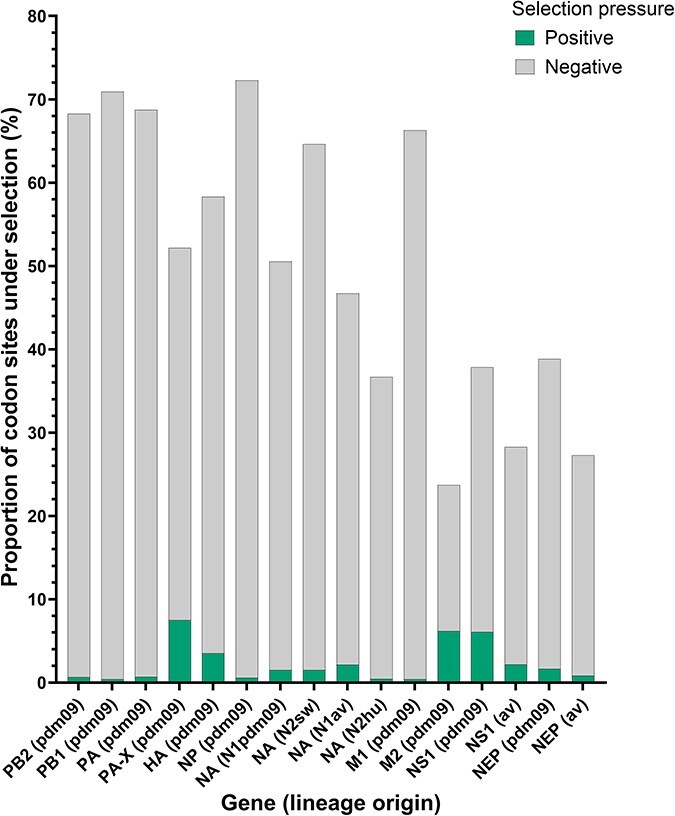
Proportion of codon sites in H1 pandemic 2009 (H1pdm09Nx) genes evolving under selective pressures as detected by FEL site-based model. Positive, diversifying selection pressure is represented in green and negative, purifying selection pressure in gray. Supporting data and additional results from other site-based selection detection models are provided in Supplementary Table 4.

Evidence of selection acting on the evolution of individual codon sites in the 11 IAV proteins was evaluated by three site-based approaches: FEL and SLAC for both positive pervasive and negative purifying selection, and MEME for both positive pervasive and positive episodic (or diversifying) selection. The results from FEL illustrate the dominance of negative purifying selection, while positive pervasive selection acted on <9% of the codon sites for any protein (Fig. 4, Supplementary Table 4). Consolidation with the other selection models indicated the genes with the largest proportion of codons subjected to negative selection are NP (64.3–72.3% per model), PB1 (63.5–70.9% per model), and PA (62.4–68.7% per model) (Supplementary Table 4). Conversely, the proteins with the largest proportion of codons that evolved under positive selection are M2 (5.2–8.3% per model), PA-X (5.1–7.5% per model), and NS1 of H1N1pdm09 origin (3.5–7.0% per model). These specific codon sites, listed for each protein in Supplementary Table 5, largely evolved under both episodic and pervasive positive selection.

### 3.5. Inheritance of zoonotic facilitating amino acid mutations

An approach to screen for amino acid mutations in swIAV with possible impacts on zoonotic ability was to compare the amino acid mutations inherited by both zoonotic IAVs, A/Denmark/1/2021(vH1N1) and A/Denmark/36/2021(vH1N1), against the amino acid mutations inherited by Danish swIAV and human seasonal H1N1pdm09 populations. The relevance of the amino acid mutation within a zoonotic context can then be speculated upon based on the presence or absence in swIAV and human seasonal H1N1pdm09 populations.

Of the 50 mutated protein sites inherited by both zoonotic IAVs, 19 (38%) were observed in human seasonal H1N1pdm09 from clade HUMS, 46 (92%) in clade DSWP from where the full genome of A/Denmark/1/2021(vH1N1) emerged, and 22 (44%) in clade N1AV from where the HA and NA segments of A/Denmark/36/2021(vH1N1) were inherited (Table 1). The proteins with the most common amino acid mutations were PB2 (*n* = 14), HA (*n* = 14), and PA (*n* = 7). Literature searches for experimental evidence of the mutational outcomes found ∼50% of the sites had not yet been clearly characterized. Amino acid mutations with an experimentally studied impact were most commonly involved in, by broad terms, immune evasion, replication, and pathogenicity. The amino acid mutations listed in Table 1 and the positively selected codon sites listed in Supplementary Table 5 overlapped by 15 protein sites: PB2-N456, PB2-I559, PA-N321, PA-I330, PA-X-A212, PA-X-R221, PA-X-R229, HA-K159, HA-S179, HA-I183, HA-S200, HA-S202, HA-S207, NP-D53, and NA-A232 (H1 numbering), indicating that the majority of the amino acid mutations inherited by the zoonotic IAVs had not evolved under positive selection.

**Table 1.** Inheritance of amino acid (AA) substitutions.

Protein	Site	Ancestral AA	AA substitutions						Impact on viral activity
			DK01	DK36	HUMS	DSWP	N1AV	REVZ	
PB2	54	R	K	K	K	K	-	-	Unknown
	76	T	A	A	-	A/T	-	-	Replication (Günl et al. 2023)
	195	D	N	N	N	N	-	-	Transmissibility (Sang et al. 2015)
	283	M	I	I	-	I	-	-	Virulence (Wang et al. 2017)
	299	R	K	K	K	K	-	-	Unknown
	344	V	M	V	M	M	-	-	Cap-binding; replication (Dipali et al. 2016, Arai et al. 2018)
	354	I	L	L	L	L	-	-	Cap-binding (Dipali et al. 2016)
	453	S	P	P	T	P	P	-	Replication (Chengzhi et al. 2020)
	*456	N	S	S	-	S	-	-	Unknown
	497	S	T	T	-	T	N	N	Unknown
	505	R	K	R	-	K	-	-	Unknown
	*559	I	V	V	-	V/I	-	-	Unknown
	588	T	I	I	-	I	I	I	Immune evasion (Zhao et al. 2014)
	590	S	N	G	-	N	-	-	Replication (Qinfang et al. 2012, Peacock et al. 2023)
PB1	317	M	I	I	-	I/V	V	-	Pathogenicity (Katz et al. 2000)
PA	212	R	L	L	-	L	-	H	Unknown
	241	C	Y	Y	-	Y	Y	-	Unknown
	269	R	K	K	-	K	-	K	Unknown
	*321	N	K	K	K	K	-	N	Pathogenicity; replication (Meng et al. 2022)
	*330	I	V	V	V	I/V	V	I	Pathogenicity; replication (Meng et al. 2022)
	350	N	D	T	-	-	-	-	Unknown
	438	I	V	V	-	I/V	-	-	Unknown
PA-X	*212	A	S	S	-	S	-	-	Unknown
	*221	R	Q	Q	Q	Q	-	-	Host shutoff (Aitor et al. 2018)
	*229	L	S	S	S	S/L	-	-	Host shutoff (Aitor et al. 2018)
HA (H1 numbering)	146	N	K	S	D	-	-	-	Unknown
	*159	K	S	S	-	D/N/S	K/N	R	Unknown
	172	G	T	E	-	A/T/V	E	E	Immune evasion; receptor binding (Ilyushina et al. 2010)
	*179	S	N	N	N	K/N	-	K	Immune evasion (Job et al. 2013, Khatib et al. 2018)
	180	K	I	I	K/Q/T	I/M	I	-	Immune evasion (Raymond et al. 2018)
	*183	I	T	V	-	F	V	-	Unknown
	*200	S	P	P	P	P	P	-	Receptor binding (Ilyushina et al. 2010, de Vries et al. 2013)
	*202	S	A	S	I	A/T	N	A	Immune evasion; receptor binding (de Vries et al. 2013)
	*207	S	W	W	-	R/W	T/W	R	Immune evasion (Gao et al. 2020)
	222	R	K	K	-	K/T	K	K	Unknown
	291	D	E	N	-	E	N	-	Unknown
	338	I	E	T	-	D/E/N/V	T	I	Unknown
	391	E	K	G	K	K/T	G	-	Unknown
	468	S	N	S	N/T	N	G	-	Fusion (Kirkpatrick et al. 2018)
NP	*53	D	E	E	-	E	-	E	Immune evasion (Mänz et al. 2013)
	100	V	I	M	-	M/V	-	M	Immune evasion (Mänz et al. 2013)
NA (N1 numbering)	67	V	I	I	-	I	I	I	Immune evasion (Wang et al. 2023)
	*232	A	V	V	-	-	V	-	Unknown (Zanin et al. 2017)
	331	K	N	G	-	N/T	G	N	Unknown
	369	N	K	K	K	K	K	-	Virulence (Abed et al. 2014)
NS1	59	L	C	H	-	-	-	-	Unknown
	101	D	N	N	-	G	-	-	Unknown
	125	E	N	N	D	D	-	-	Host shutoff (Clark et al. 2017)
	137	I	V	V	-	L/T	-	-	Unknown
NEP	60	S	N	N	-	N	-	-	Unknown

AA sequence of A/California/07/2009 represents an early ancestor of the H1N1 pandemic 2009 (H1N1pdm09) lineage (ancestral AA). AA substitutions inherited by both zoonotic viruses, A/Denmark/1/2021 (DK01) and A/Denmark/36/2021 (DK36), are compared against AA substitutions inherited by human seasonal H1N1pdm09 in clade HUMS (HUMS), swine H1pdm09Nx phylogenetic clades (DSWP and N1AV), and reverse zoonotic viruses (REVZ) inherited from the last common ancestor in clade HUMS. Multiple AA substitutions at identical protein sites (/) and absence of AA substitutions (-) are indicated. Possible impacts of AA substitutions on viral activity were inferred from published experimental studies. To note, H1 numbering of protein sites begins from first methionine, but referenced studies may use H3 numbering or H1 numbering starting from asparagine 18. Protein sites that also evolved under positive selection are indicated (*).

### 3.6. Immune evasion by post-translational glycosylation of HA protein

**Figure 5.**
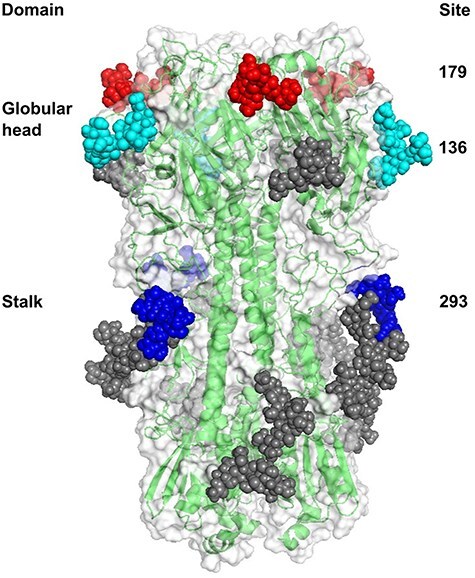
Structural representation of an HA homotrimer based on A/Denmark/36/2021(vH1N1) with N-linked glycans attached to predicted N-linked glycosylation HA protein sites. Glycans were rendered as spheres and colored to highlight glycans at HA sites (H1 numbering) 179 in red, 136 in cyan, and 293 in blue (glycans at sites 28, 40, 104, 304, and 498 were shaded in gray). Supporting data are provided in Supplementary Table 6.

Evading pre-existing immunity is an important step to effectively initiate infection in a new host and one mechanism exploited by IAV is the post-translational attachment of glycans on the HA protein to disguise antigenic sites from antibody recognition. Asparagine (N)-linked glycosylation sites were predicted on the H1pdm09Nx HA amino acid sequences and visualized on a HA homotrimer model (Fig. 5). In contrast to the glycosylation profile of early 2009 H1N1pdm09, the most noticeable glycosylation sites gained in the H1pdm09Nx populations were at positions 136, 179, and 293 (H1 numbering). Of these, only one glycosylation site at position 179 in the globular head region had become prominent in human seasonal H1N1pdm09 after the 2016/2017 season, following the amino acid substitution of S179N. This substitution was also present in 67% of the reverse zoonotic, human seasonal-like IAVs in Danish swine farms, as well as both zoonotic IAVs (A/Denmark/01/2021(vH1N1) and A/Denmark/36/2021(vH1N1)) and between 10%and 56% of Danish swIAV H1pdm09Nx populations (Supplementary Table 4). Glycosylation site at position 136 was predicted for only one of the two zoonotic IAVs (A/Denmark/36/2021(vH1N1)) and the vast majority of swIAV H1pdm09N1av from which A/Denmark/36/2021(vH1N1) descends, following the amino acid substitution of K136N. A third possible glycosylation site at 293 was predicted on both zoonotic IAVs, all Danish swIAV H1pdm09N1av, and 48% of Danish swIAV H1N1pdm09, despite the conservation of the glycosylation motif, N-X-S/T, at positions 293 to 295 across the entire phylogeny. Another noteworthy glycosylation site at position 202 was predicted for 94% of Danish swIAV H1pdm09N1av, yet A/Denmark/36/2021(vH1N1) had a substitution of N202S that disrupted the glycosylation site.

## 4. Discussion

Genetically diverse populations of H1pdm09Nx with zoonotic potential continue to evolve in Danish swine farms. This study examined the evolutionary dynamics of H1pdm09Nx from whole-genome sequencing data in an effort to deduce viral genetic indicators of host adaptation and pig–human transmission.

The H1N1pdm09 ancestors of Danish swIAV H1pdm09Nx had already begun diverging in late 2008, while the virus was presumably circulating undetected among humans for several months after the initial zoonotic jump (Kelly et al. 2010, Mena et al. 2016). During the early waves of pandemic outbreaks, multiple genetic variants of H1N1pdm09 spilled over into European and Danish pig farms and differentiated into the initial Danish swIAV clades by approximately late 2010, according to the molecular clock analysis and other reports (Watson et al. 2015). Reverse zoonosis of H1N1pdm09 proceeded over subsequent seasonal epidemics but failed to establish new lineages in Danish swine, causing only isolated infections in pigs. Similarly, the authors of a study examining the phylodynamics of H1N1pdm09 in swine farms in Brazil reported mostly “dead-end” reverse zoonotic introductions after 2012 (Junqueira et al. 2023). This loss of ability for human seasonal H1N1pdm09 to become enzootic swIAVs coincides with an evolutionary shift observed in the human population; immune escape replaced host adaptation as the major evolutionary driver, prompting successive seasonal bottlenecks that culminated in the large-scale reduction of genetic diversity in 2014 (Su et al. 2015).

Contrarily, the genetic diversity of H1N1pdm09 expanded across Danish swine farms. The full H1N1pdm09 genome persists in several subclusters that had descended from a single common ancestor in clade DSWP. Lineage-specific, positive selection detected on the initiating branch of clade DWSP in the HA gene phylogeny is consistent with IAV adaptation to a new host species (Smith et al. 2015). Along this branch, 78% of the amino acid substitutions occurred inside the receptor-binding domain (H1 sites 113–265; Ghafoori et al. 2023), which hosts several antigenic sites, suggesting that this primary antigenic drift likely contributed to the sustained transmission of nonreassorted H1N1pdm09 in Danish swine. Reassortments of H1N1pdm09 with NA segments from enzootic H1avN2sw (typically the dominant subtype lineage infecting Danish swine farms; Ryt-Hansen et al. 2021) occurred as early as 2010 and prompted the formation of additional H1pdm09Nx populations, further accelerating the genetic diversity. A later NA segment reassortment event between an ancestor of H1pdm09N2sw in clade N2PA and H1N1av likely occurred after 2011 to create the H1pdm09N1av lineage first detected in early 2018 (Ryt-Hansen et al. 2021). During the same time frame, the HA gene in clade N1AV also experienced positive, lineage-specific selection and substantial genetic drift, possibly to accommodate the new NA gene as the functions of the two viral envelope glycoproteins are fundamentally linked. HA binds and NA cleaves sialic acids, and proportionate HA–NA activity was required for effective airborne transmission of H1N1pdm09 swIAVs (demonstrated in the ferret model of human transmission), which likely primed the virus for the 2009 pandemic (Lakdawala et al. 2011; Xu et al. 2012, Zanin et al. 2015). Even though N1pdm09 and N1av genomic segments originate from the 1970s H1N1av lineage, both had undergone considerable genetic divergence since pre-2009 ancestral H1N1av with potential implications for the functional balance of HA–NA.

The capacity for multiple H1pdm09Nx lineages to cocirculate in Danish swine farms is partly attributed to the immunogenicity of the various NA proteins (N1 and N2 subtypes occupy separate phylogenetic groups; Russell et al. 2006) and pig production practices involving high turnovers of pigs that impede the development of herd immunity (Pitzer et al. 2016). In a study examining H1N1pdm09 in swine farms in Germany, two major clades were defined that overlap with two Danish swIAV H1pdm09Nx clades: “Papenburg/2010-like H1pdmN2” (containing the German H1pdm09Nx predecessor of clade N2PA) and “Wachtum/2014-like H1pdmN1pdm” (containing German H1pdm09Nx that clustered within clade DSWP) (Zell et al. 2020). The antigenic cross-reactivity between these German swIAVs, as assessed by hemagglutination inhibition (HI) test with hyperimmune sera generated in pigs, was reportedly low. Extrapolation of these results to Danish swine farms would suggest that a prior infection with H1N1pdm09 from clade DSWP would generate antibodies in pigs with limited cross-immunity protection against a subsequent encounter with H1pdm09N2sw from clade N2PA, thus perpetuating the transmission chains of multiple H1pdm09Nx lineages. Appreciably, the analyses in this study compiles the sequences of swIAVs sampled from swine farms across Denmark and thus represents the collective viral genetic diversity. Individual swine farms can essentially be regarded as isolated pig populations that enable independent viral evolution with considerable antigenic drift as previously identified (Ryt-Hansen et al. 2020). Therefore, the observed genetic diversity of the H1pdm09Nx populations in this study is most likely derived from between-farm variation than within-farm variation. In addition, mass sow vaccination programs with whole-inactivated viruses can further exacerbate genetic drift of the HA protein (Ryt-Hansen et al. 2019, López-Valiñas et al. 2023). Vaccination with the commercially available and widely used Respiporc FLU3 vaccine (based on three pre-2009 swIAV strains) in Danish swine farms impacted genetic drift and reinfection of H1N1av and H1avN2sw swIAVs (Ryt-Hansen et al. 2019, Bhatta et al. 2020). The vaccine against H1N1pdm09 (Respiporc FLUpan) was approved for use in 2017, but the direct impacts on H1pdm09Nx evolution are unknown.

Approximately 11 years after the divergence from human seasonal H1N1pdm09, the first two detections of zoonotic IAVs emerged from Danish swIAV H1pdm09Nx. Results of HI tests presented in the case reports showed weak antigenic cross-reactivity of the human seasonal H1N1pdm09 vaccine against the zoonotic IAVs (Nissen et al. 2021, Andersen et al. 2022), indicating that the human population would probably possess low levels of pre-existing immunity (however, the antisera used in the HI tests was generated in ferrets which is not a representative of human lifetime exposure to IAVs; Hensley 2014). In the present study, predictions of N-linked glycosylation revealed the addition of two potential glycosylation sites on the HA proteins of Danish swIAV H1pdm09Nx that were absent in human seasonal H1N1pdm09. The attachment of a glycan at HA 136N to A/Denmark/36/2021(vH1N1) and related H1pdm09Nx in clade N1AV was associated with resistance to neutralizing antibodies against human seasonal H1N1pdm09-vaccinated mice (Job et al. 2013), and a predicted glycan at position 293N in the stalk region of HA proteins from both zoonotic IAVs and Danish swIAV H1pdm09Nx has not been previously described. The HA proteins of both zoonotic IAVs also accrued the most amino acid mutations in the receptor-binding region and antigenic sites, which likely enhanced the ability to evade antibody recognition and initiate infection in the humans. Circumstantially, the two zoonotic IAVs emerged under COVID-19 restrictions that prevented a typical influenza epidemic season and resulted in huge reductions of genetic diversity in human seasonal H1N1pdm09 and other human seasonal IAVs (Dhanasekaran et al. 2022). The impacts of this low genetic diversity might increase the vulnerability of the human population to future pandemics, as strong immunological biases mounted against the limited diversity of current human seasonal IAVs might prevent broader cross-immunity protection against emerging zoonotic IAV threats (Gostic et al. 2019).

Most of the zoonotic IAV segments, as well as the founding IAVs of some H1pdm09Nx clades, particularly clade N1AV, appear at the end of long phylogenetic branches. These time frames spanning several years uninterrupted from reconstructed ancestor to descendant IAV can allude to undersampling of the viral population. Indeed, a large proportion of H1pdm09Nx submissions were not recovered in this study, and the Danish swIAV surveillance operates passively, only receiving submissions from swine farms displaying influenza illness, and consequently it lacks the swIAVs that cause asymptomatic infections. Alternatively, the branches of the phylogenetic molecular clock trees were scaled by the same, global evolutionary rate. The *χ*^2^ and *r*^2^ scores indicated a fluctuation of rates across the phylogeny; therefore, increases in evolutionary rate can elongate the branches by placing reconstructed ancestors at an earlier date to compensate for larger than expected genetic distances of the sampled descendent virus. Regardless, the dates of clade divergence inferred in this study provide an adequate indication of the relationships between reconstructed ancestors and sampled descendant viruses across the phylogeny. The inclusion of clade HUMS in the molecular clock rate estimations likely contributed to the rate variation, as the clade encompasses human seasonal H1N1pdm09 isolated mainly from humans and a few incidental swine, but serves to capture host adaptation of Danish swIAVs in contrast to human seasonal IAVs (i.e. a gene undergoing extensive genetic changes in one host population but not the other is relevant for identifying markers of host adaptation).

Genes evolving under higher negative purifying selection, i.e. PB2, PB1, PA, NP, and M1, likely exhibit stronger evolutionary constraints, suggesting nucleotide mutations that arise and become fixed could be significantly advantageous to viral fitness in the host. Inversely, the association of slower segment evolutionary rates with larger proportions of codon sites evolving under positive selection, i.e. NS1, M2, and PA-X, could be interpreted as the genes evolving more “determinedly” to adapt to critical changes in the host environment; however, genes with overlapping reading frames can lead to inflated selection measurements due to their inherent evolutionary constraints (Pavesi 2021). Curiously, the two zoonotic IAVs emerged from two separate reassortant populations of H1pdm09Nx with NSav segments that first appeared in 2020 (a single reassortment of this type was detected in 2017 but further descendants were not recovered). Moreover, the internal gene cassette of H1N1pdm09 origin had remained in a stable configuration until these reassortment events. A function of NS1 intersects with the function of PA-X to suppress general host gene expression, which inevitably restricts antiviral responses and facilitates IAV replication (Levene and Maria Gaglia 2018). Early pandemic H1N1pdm09 controlled human gene expression with PA-X, while NS1 was ineffective (Hale et al. 2010, Hayashi et al. 2016), but after several epidemic seasons, NS1 accumulated amino acid mutations that restored the host shutoff ability and gained dominant control from PA-X (Aitor et al. 2018). How this activity is coordinated between the two proteins in H1pdm09Nx that infect pigs, however, remains unknown.

Other prominent commonalities between the two zoonotic IAVs are the shared amino acid mutations in the PB2 protein. Since the MRCA in late 2014, both zoonotic IAVs separately acquired amino acid mutations PB2-S590N/G and PB2-T588I. In combination with PB2-271A and PB2-591R, PB2-590S was essential for H1N1pdm09 to replicate in mammalian cells, as the PB2 gene of H1N1pdm09 was originally derived from an avian IAV spillover (Qinfang et al. 2012). Therefore, assessing the effect of PB2-590N/G on the replicative efficiency of H1N1pdm09 in human cells would provide valuable insight for the transmission of zoonotic IAVs. The PB2-588I mutation can antagonize the interferon pathway of the innate immune system and enhance the virulence in cells and mice models (Zhao et al. 2014). Accordingly, measuring the expression of the innate immune pathways and the synthesis of antiviral proteins in human cells would help predict the potential consequences of human infection with zoonotic IAVs.

In conclusion, this study provides a close examination on the evolution of H1N1pdm09 descendant IAVs across Danish swine farms that led to two zoonotic infections. The lists of inherited amino acid mutations and codon sites evolving under positive selection point to candidate viral markers of host adaptation and zoonotic potential for further characterization.

## Supplementary Material

veaf014_Supp

## Data Availability

Data are available at https://doi.org/10.5281/zenodo.13794353 and include a list of all IAV isolates in the sequencing dataset, segment sequence alignments, IQ-TREE log files and conTree output, selected TreeTime analysis output, and phylogenetic molecular clock trees with annotated amino acid mutations.
