# Supplementary material for: Evolutionary dynamics and molecular epidemiology of H1N1 pandemic 2009 influenza A viruses across swine farms in Denmark
Source: Virus Evol. 2025 Mar 7;11(1):veaf014. doi: 10.1093/ve/veaf014 (PMC11997423; doi:10.1093/ve/veaf014)
Supplement: veaf014_Supp [file veaf014_supp.zip › supplementary.docx]

**11. Supplementary figures and tables**

**Supplementary Table 1)** List of H1 pandemic 2009 (H1pdm09, H1 clade 1A.3.3.2) influenza A viruses (IAVs) detected in Danish swine farms and zoonotic IAVs (*) included in the sequence dataset. The full list of all IAVs analyzed is available in **Supporting Files** (isolates.xlsx). Accession and isolate IDs (prefixed by “A/swine/Denmark/”) are recorded in GISAID and GenBank sequence databases. Referenced accession numbers indicate the inclusion of the sequence in previous analysis. Isolates were numbered and renamed with subtype-lineage classification for the phylogenetic molecular clock trees in **Figure 2**. NA lineages are classified based on origin as “N1pdm09” from H1N1pdm09, “N1av” from 1970s H1N1 Eurasian avian, “N2sw” from A/swine/Gent/84 H3N2-like and “N2hu” from 1990s human seasonal H3N2-like IAVs. Genomic segments of each isolate were assigned to defined phylogenetic clades (DSWP, HUMS, N1AV, N2HU and N2PA) and segments without designations did not cluster within any of the defined clades (n/a) or were absent from the reconstructed genome and phylogeny (-). NS segments from H1N1av origin are indicated as “AV”.

| **Accession number** | **Sample ID/ collection year** | **Phylogenetic tree tip label** | **NA lineage** | **Phylogenetic clade assignment** | | | | | | | |  |
| --- | --- | --- | --- | --- | --- | --- | --- | --- | --- | --- | --- | --- |
|  |  |  |  | **PB2** | **PB1** | **PA** | **HA** | **NP** | **NA** | **M** | **NS** | |
| PQ192919-PQ192926 | 10-50-16/  2010 | 131-H1AN2H | N2hu | N2HU | N2HU | N2HU | N2HU | N2HU | N2HU | n/a | N2HU | |
| EPI_ISL_195134  (Watson et al. 2015) | 10-1310-1/  2011 | 112-H1AN1P | N1pdm09 | n/a | n/a | n/a | n/a | n/a | n/a | N2PA | n/a | |
| PQ192779-PQ192783 | 11-1568-1/  2011 | 132-H1AN2H | N2hu | - | - | - | N2HU | N2HU | N2HU | N2HU | N2HU | |
| EPI_ISL_195048  (Watson et al. 2015) | 10-2779-2/  2011 | 115-H1AN1P | N1pdm09 | n/a | n/a | n/a | n/a | DSWP | n/a | n/a | - | |
| PQ192784-PQ192790 | 12-10-162-1/  2012 | 133-H1AN1P | N1pdm09 | n/a | - | n/a | n/a | n/a | n/a | n/a | n/a | |
| PQ192791-PQ192796 | 12-10-176-1/  2012 | 134-H1AN2D | N2sw | - | - | n/a | n/a | n/a | n/a | n/a | n/a | |
| EPI_ISL_179708  (Andersen et al. 2022) | 10845-1/  2012 | 125-H1AN2D | N2sw | n/a | n/a | N2PA | n/a | n/a | n/a | N2PA | n/a | |
| EPI_ISL_4081562-EPI_ISL_4081563  (Ryt-Hansen et al. 2021) | 2013_10_28_4p1/  2013 | 146-H1AN1P | N1pdm09 | n/a | - | n/a | n/a | n/a | n/a | n/a | - | |
| PQ192967-PQ192974 | 13-519-1/  2013 | 155-H1AN1P | N1pdm09 | n/a | n/a | n/a | n/a | n/a | n/a | n/a | n/a | |
| EPI_ISL_4081570-EPI_ISL_4081571  (Ryt-Hansen et al. 2021) | 2013_10_685_1p1/  2013 | 147-H1AN1P | N1pdm09 | n/a | - | n/a | n/a | DSWP | n/a | n/a | n/a | |
| PQ192927-PQ192934 | 13-1070-1/  2013 | 149-H1AN2D | N2sw | n/a | n/a | n/a | n/a | n/a | n/a | n/a | n/a | |
| EPI_ISL_4081560-EPI_ISL_4081561  (Ryt-Hansen et al. 2021) | 2013_10_1325_5p1/  2013 | 138-H1AN2D | N2sw | n/a | n/a | n/a | n/a | n/a | n/a | - | n/a | |
| PQ192797-PQ192803 | 13-1329-2/  2013 | 150-H1AN2D | N2sw | n/a | n/a | n/a | n/a | n/a | n/a | - | n/a | |
| PQ192935-PQ192942 | 13-1413-1/  2013 | 151-H1AN1P | N1pdm09 | n/a | n/a | n/a | n/a | DSWP | n/a | n/a | n/a | |
| PQ192943-PQ192950 | 13-1495-1/  2013 | 152-H1AN1P | N1pdm09 | n/a | n/a | n/a | n/a | n/a | n/a | n/a | n/a | |
| PQ192951-PQ192958 | 13-2361-1/  2013 | 153-H1AN2D | N2sw | n/a | n/a | n/a | n/a | n/a | n/a | DSWP | n/a | |
| PQ192959-PQ192966 | 13-2458-2/  2013 | 154-H1AN1P | N1pdm09 | n/a | n/a | n/a | n/a | DSWP | n/a | n/a | n/a | |
| PQ193039-PQ193046 | 14-188-8/  2014 | 178-H1AN1P | N1pdm09 | n/a | n/a | n/a | n/a | n/a | n/a | n/a | n/a | |
| EPI_ISL_4081542-EPI_ISL_4081543  (Ryt-Hansen et al. 2021) | 2014_10_203_1p1/  2014 | 156-H1AN2H | N2hu | N2HU | N2HU | N2HU | N2HU | N2HU | N2HU | N2HU | N2HU | |
| EPI_ISL_4081572-EPI_ISL_4081573  (Ryt-Hansen et al. 2021) | 2014_10_231_1p1/  2014 | 157-H1AN1P | N1pdm09 | n/a | n/a | n/a | n/a | DSWP | n/a | n/a | n/a | |
| EPI_ISL_4081544-EPI_ISL_4081545  (Ryt-Hansen et al. 2021) | 2014_10_329_2p1/  2014 | 161-H1AN2H | N2hu | N2HU | N2HU | N2HU | N2HU | N2HU | N2HU | N2HU | N2HU | |
| PQ193055-PQ193062 | 14-365-3/  2014 | 180-H1AN1P | N1pdm09 | n/a | n/a | n/a | n/a | n/a | n/a | n/a | n/a | |
| PQ193071-PQ193078 | 14-500-1/  2014 | 182-H1AN1P | N1pdm09 | DSWP | DSWP | DSWP | DSWP | DSWP | DSWP | DSWP | DSWP | |
| PQ192975-PQ192982 | 14-1006-2/  2014 | 164-H1AN1P | N1pdm09 | DSWP | DSWP | DSWP | DSWP | DSWP | DSWP | DSWP | DSWP | |
| PQ193063-PQ193070 | 14-4-1/  2014 | 181-H1AN1P | N1pdm09 | DSWP | n/a | DSWP | DSWP | DSWP | DSWP | DSWP | DSWP | |
| PQ193047-PQ193054 | 14-242-2/  2014 | 179-H1AN2D | N2sw | n/a | n/a | n/a | DSWP | n/a | n/a | n/a | n/a | |
| PQ193079-PQ193086 | 14-6252-2/  2014 | 183-H1AN2D | N2sw | n/a | n/a | N2PA | n/a | n/a | n/a | n/a | n/a | |
| EPI_ISL_4081534-EPI_ISL_4081535  (Ryt-Hansen et al. 2021) | 2014_9477_1p1_1/  2014 | 185-H1AN1P | N1pdm09 | DSWP | DSWP | DSWP | DSWP | DSWP | n/a | DSWP | DSWP | |
| EPI_ISL_4081546-EPI_ISL_4081547  (Ryt-Hansen et al. 2021) | 2014_10781_1p1/  2014 | 165-H1AN2D | N2sw | N2PA | N2PA | N2PA | N2PA | N2PA | N2PA | N2PA | N2PA | |
| PQ192983-PQ192990 | 14-12310-1/  2014 | 166-H1AN1P | N1pdm09 | n/a | n/a | n/a | n/a | n/a | n/a | n/a | n/a | |
| PQ192991-PQ192998 | 14-13336-1/  2014 | 167-H1AN1P | N1pdm09 | n/a | n/a | n/a | n/a | DSWP | n/a | n/a | n/a | |
| PQ192999-PQ193006 | 14-13671-1/  2014 | 168-H1AN1P | N1pdm09 | n/a | n/a | n/a | n/a | DSWP | n/a | n/a | n/a | |
| PQ193007-PQ193014 | 14-13678-1/  2014 | 169-H1AN1P | N1pdm09 | HUMS | HUMS | n/a | HUMS | HUMS | HUMS | HUMS | HUMS | |
| PQ193015-PQ193022 | 14-14008-1/  2014 | 170-H1AN1P | N1pdm09 | DSWP | DSWP | DSWP | DSWP | DSWP | DSWP | DSWP | DSWP | |
| PQ192804-PQ192810 | 14-14831-1/  2014 | 171-H1AN2D | N2sw | N2PA | N2PA | N2PA | n/a | N2PA | N2PA | N2PA | N2PA | |
| PQ192811-PQ192817 | 14-15244-1/  2014 | 173-H1AN1P | N1pdm09 | - | DSWP | DSWP | DSWP | DSWP | DSWP | DSWP | DSWP | |
| PQ193023-PQ193030 | 14-15404-1/  2014 | 174-H1AN1P | N1pdm09 | DSWP | DSWP | DSWP | DSWP | DSWP | DSWP | DSWP | DSWP | |
| PQ193031-PQ193038 | 14-15831-1/  2014 | 175-H1AN1P | N1pdm09 | n/a | n/a | n/a | n/a | n/a | n/a | n/a | n/a | |
| PQ193231-PQ193238 | 15-729-2/  2015 | 223-H1AN1P | N1pdm09 | DSWP | n/a | DSWP | DSWP | DSWP | DSWP | DSWP | DSWP | |
| PQ193263-PQ193270 | 15-775-1/  2015 | 227-H1AN1P | N1pdm09 | DSWP | n/a | DSWP | DSWP | DSWP | DSWP | DSWP | DSWP | |
| EPI_ISL_4081554-EPI_ISL_4081555  (Ryt-Hansen et al. 2021) | 2015_00798_2p1/  2015 | 186-H1AN2H | N2hu | n/a | n/a | n/a | n/a | DSWP | n/a | n/a | n/a | |
| EPI_ISL_4081548-EPI_ISL_4081549  (Ryt-Hansen et al. 2021) | 2015_03627_2p1/  2015 | 189-H1AN2D | N2sw | N2PA | N2PA | n/a | N2PA | N2PA | N2PA | N2PA | N2PA | |
| PQ193183-PQ193190 | 15-3655-1/  2015 | 215-H1AN1P | N1pdm09 | DSWP | DSWP | DSWP | DSWP | DSWP | DSWP | DSWP | DSWP | |
| PQ193191-PQ193198 | 15-3984-1/  2015 | 216-H1AN1P | N1pdm09 | DSWP | DSWP | DSWP | DSWP | DSWP | DSWP | DSWP | DSWP | |
| PQ193199-PQ193206 | 15-4480-1/  2015 | 217-H1AN1P | N1pdm09 | DSWP | n/a | DSWP | DSWP | DSWP | DSWP | DSWP | DSWP | |
| EPI_ISL_4081556-EPI_ISL_4081557  (Ryt-Hansen et al. 2021) | 2015_04804_3p1/  2015 | 192-H1AN2D | N2sw | n/a | n/a | N2PA | n/a | n/a | n/a | n/a | n/a | |
| PQ193207-PQ193214 | 15-5736-1/  2015 | 218-H1AN1P | N1pdm09 | DSWP | DSWP | DSWP | DSWP | DSWP | DSWP | DSWP | DSWP | |
| EPI_ISL_4081528-EPI_ISL_4081529  (Ryt-Hansen et al. 2021) | 2015_05775_2p1/  2015 | 194-H1AN1P | N1pdm09 | DSWP | DSWP | DSWP | DSWP | DSWP | DSWP | DSWP | DSWP | |
| PQ193215-PQ193222 | 15-5774-2/  2015 | 220-H1AN1P | N1pdm09 | DSWP | DSWP | DSWP | DSWP | DSWP | DSWP | DSWP | DSWP | |
| PQ193223-PQ193230 | 15-6702-3/  2015 | 221-H1AN1P | N1pdm09 | DSWP | DSWP | DSWP | DSWP | DSWP | DSWP | DSWP | DSWP | |
| PQ192831-PQ192835 | 15-6829-2/  2015 | 222-H1AN1P | N1pdm09 | - | - | - | HUMS | HUMS | HUMS | HUMS | HUMS | |
| PQ193239-PQ193246 | 15-7570-1/  2015 | 224-H1AN1P | N1pdm09 | DSWP | DSWP | DSWP | DSWP | DSWP | DSWP | DSWP | DSWP | |
| PQ193247-PQ193254 | 15-7689-1/  2015 | 225-H1AN1P | N1pdm09 | DSWP | DSWP | DSWP | DSWP | DSWP | DSWP | DSWP | DSWP | |
| PQ193255-PQ193262 | 15-7748-1/  2015 | 226-H1AN1P | N1pdm09 | DSWP | DSWP | DSWP | DSWP | DSWP | DSWP | DSWP | DSWP | |
| PQ192825-PQ192830 | 15-5740-2/  2015 | 219-H1AN2D | N2sw | - | - | n/a | n/a | DSWP | n/a | n/a | n/a | |
| PQ193271-PQ193278 | 15-8139-1/  2015 | 228-H1AN1P | N1pdm09 | DSWP | DSWP | DSWP | DSWP | DSWP | DSWP | DSWP | DSWP | |
| PQ193279-PQ193286 | 15-8641-1/  2015 | 229-H1AN1P | N1pdm09 | DSWP | DSWP | DSWP | DSWP | DSWP | DSWP | DSWP | DSWP | |
| PQ193287-PQ193294 | 15-8648-1/  2015 | 230-H1AN2D | N2sw | N2PA | N2PA | N2PA | n/a | N2PA | N2PA | N2PA | N2PA | |
| PQ193295-PQ193302 | 15-8890-1/  2015 | 231-H1AN1P | N1pdm09 | DSWP | DSWP | DSWP | DSWP | DSWP | DSWP | DSWP | DSWP | |
| PQ192836-PQ192842 | 15-9446-2/  2015 | 232-H1AN1P | N1pdm09 | n/a | - | n/a | n/a | DSWP | n/a | n/a | n/a | |
| PQ193303-PQ193310 | 15-9588-1/  2015 | 233-H1AN1P | N1pdm09 | DSWP | DSWP | DSWP | DSWP | DSWP | DSWP | DSWP | DSWP | |
| EPI_ISL_4081540-EPI_ISL_4081541  (Ryt-Hansen et al. 2021) | 2015_10377_1p1/  2015 | 197-H1AN2H | N2hu | N2HU | N2HU | N2HU | N2HU | N2HU | N2HU | N2HU | N2HU | |
| PQ193087-PQ193094 | 15-12237-1/  2015 | 198-H1AN1P | N1pdm09 | n/a | n/a | N2PA | n/a | n/a | n/a | N2PA | n/a | |
| PQ193095-PQ193102 | 15-12515-1/  2015 | 199-H1AN1P | N1pdm09 | DSWP | HUMS | DSWP | DSWP | DSWP | DSWP | DSWP | DSWP | |
| EPI_ISL_216578  (Henritzi et al. 2020) | AR1855/  2015 | 405-H1AN1P | N1pdm09 | DSWP | DSWP | DSWP | DSWP | DSWP | DSWP | DSWP | DSWP | |
| PQ193103-PQ193110 | 15-14309-1/  2015 | 200-H1AN1P | N1pdm09 | DSWP | DSWP | DSWP | DSWP | DSWP | DSWP | DSWP | DSWP | |
| PQ193111-PQ193118 | 15-14619-1/  2015 | 201-H1AN1P | N1pdm09 | DSWP | DSWP | DSWP | DSWP | DSWP | DSWP | DSWP | DSWP | |
| PQ193119-PQ193126 | 15-18066-2/  2015 | 202-H1AN1P | N1pdm09 | DSWP | DSWP | DSWP | DSWP | DSWP | DSWP | DSWP | DSWP | |
| PQ193127-PQ193134 | 15-18092-1/  2015 | 203-H1AN2D | N2sw | n/a | n/a | N2PA | n/a | n/a | n/a | n/a | n/a | |
| PQ193135-PQ193142 | 15-18609-2/  2015 | 204-H1AN1P | N1pdm09 | n/a | n/a | n/a | n/a | n/a | n/a | N2PA | n/a | |
| PQ193143-PQ193150 | 15-18706-1/  2015 | 205-H1AN1P | N1pdm09 | DSWP | DSWP | DSWP | DSWP | DSWP | DSWP | DSWP | DSWP | |
| PQ193151-PQ193158 | 15-19087-1/  2015 | 206-H1AN1P | N1pdm09 | DSWP | DSWP | DSWP | DSWP | DSWP | DSWP | DSWP | DSWP | |
| PQ193159-PQ193166 | 15-19090-1/  2015 | 207-H1AN1P | N1pdm09 | n/a | n/a | n/a | n/a | DSWP | n/a | n/a | n/a | |
| EPI_ISL_4081530-EPI_ISL_4081531  (Ryt-Hansen et al. 2021) | 2015_19295_1p1/  2015 | 208-H1AN1P | N1pdm09 | DSWP | DSWP | DSWP | DSWP | DSWP | DSWP | DSWP | DSWP | |
| EPI_ISL_4081448-EPI_ISL_4081449  (Ryt-Hansen et al. 2021) | 2015_20566_1p1/  2015 | 209-H1AN2D | N2sw | DSWP | DSWP | DSWP | DSWP | DSWP | n/a | DSWP | DSWP | |
| PQ193167-PQ193174 | 15-20835-1/  2015 | 210-H1AN1P | N1pdm09 | DSWP | DSWP | DSWP | DSWP | DSWP | DSWP | DSWP | DSWP | |
| PQ192818-PQ192824 | 15-20887-2/  2015 | 211-H1AN1P | N1pdm09 | DSWP | n/a | DSWP | DSWP | DSWP | DSWP | DSWP | - | |
| PQ193175-PQ193182 | 15-22393-1/  2015 | 212-H1AN1P | N1pdm09 | DSWP | DSWP | DSWP | DSWP | DSWP | DSWP | DSWP | DSWP | |
| EPI_ISL_4081476-EPI_ISL_4081477  (Ryt-Hansen et al. 2021) | 2015_23655_1p1/  2015 | 214-H1AN1P | N1pdm09 | DSWP | DSWP | DSWP | DSWP | DSWP | DSWP | DSWP | DSWP | |
| PQ193351-PQ193358 | 16-208-2/  2016 | 258-H1AN1P | N1pdm09 | DSWP | DSWP | DSWP | DSWP | DSWP | DSWP | DSWP | DSWP | |
| EPI_ISL_304064  (Henritzi et al. 2020) | AR306/  2016 | 406-H1AN1P | N1pdm09 | - | - | - | DSWP | - | DSWP | - | - | |
| EPI_ISL_4081532-EPI_ISL_4081533  (Ryt-Hansen et al. 2021) | 2016_321_1p1/  2016 | 261-H1AN1P | N1pdm09 | DSWP | DSWP | DSWP | DSWP | DSWP | DSWP | DSWP | DSWP | |
| PQ193383-PQ193390 | 16-391-1/  2016 | 265-H1AN1P | N1pdm09 | DSWP | DSWP | DSWP | DSWP | DSWP | DSWP | DSWP | DSWP | |
| PQ193311-PQ193318 | 16-1521-1/  2016 | 238-H1AN1P | N1pdm09 | DSWP | DSWP | DSWP | DSWP | DSWP | DSWP | DSWP | DSWP | |
| PQ192843-PQ192849 | 16-1651-1/  2016 | 244-H1AN2D | N2sw | N2PA | N2PA | N2PA | N2PA | N2PA | N2PA | N2PA | - | |
| PQ193327-PQ193334 | 16-1673-3/  2016 | 245-H1AN1P | N1pdm09 | DSWP | DSWP | DSWP | DSWP | DSWP | DSWP | DSWP | DSWP | |
| PQ193367-PQ193374 | 16-3873-3/  2016 | 262-H1AN1P | N1pdm09 | DSWP | DSWP | DSWP | DSWP | DSWP | DSWP | DSWP | DSWP | |
| PQ193375-PQ193382 | 16-3901-3/  2016 | 264-H1AN1P | N1pdm09 | DSWP | DSWP | DSWP | DSWP | DSWP | DSWP | DSWP | DSWP | |
| EPI_ISL_4081464-EPI_ISL_4081465  (Ryt-Hansen et al. 2021) | 2016_3920_3p1/  2016 | 266-H1AN1P | N1pdm09 | DSWP | DSWP | DSWP | DSWP | HUMS | DSWP | DSWP | DSWP | |
| EPI_ISL_4081566-EPI_ISL_4081567  (Ryt-Hansen et al. 2021) | 2016_3929_1p1/  2016 | 267-H1AN1P | N1pdm09 | HUMS | HUMS | HUMS | HUMS | HUMS | HUMS | HUMS | HUMS | |
| PQ193391-PQ193398 | 16-4973-1/  2016 | 269-H1AN1P | N1pdm09 | DSWP | DSWP | DSWP | DSWP | DSWP | DSWP | DSWP | DSWP | |
| PQ193399-PQ193406 | 16-6503-2/  2016 | 270-H1AN2D | N2sw | N2PA | N2PA | n/a | N2PA | N2PA | N2PA | N2PA | N2PA | |
| PQ193407-PQ193414 | 16-9154-3/  2016 | 271-H1AN1P | N1pdm09 | N2PA | n/a | HUMS | HUMS | n/a | HUMS | N2PA | n/a | |
| EPI_ISL_4081472-EPI_ISL_4081473  (Ryt-Hansen et al. 2021) | 2016_10130_1p1/  2016 | 235-H1AN1P | N1pdm09 | DSWP | DSWP | DSWP | DSWP | DSWP | DSWP | DSWP | DSWP | |
| EPI_ISL_4081458-EPI_ISL_4081459  (Ryt-Hansen et al. 2021) | 2016_12781_1p1/  2016 | 237-H1AN1P | N1pdm09 | DSWP | DSWP | DSWP | DSWP | DSWP | DSWP | DSWP | DSWP | |
| PQ193319-PQ193326 | 16-16219-1/  2016 | 242-H1AN1P | N1pdm09 | DSWP | DSWP | DSWP | DSWP | DSWP | DSWP | DSWP | DSWP | |
| EPI_ISL_4081470-EPI_ISL_4081471  (Ryt-Hansen et al. 2021) | 2016_16988_3p1/  2016 | 247-H1AN1P | N1pdm09 | DSWP | DSWP | DSWP | DSWP | DSWP | DSWP | DSWP | DSWP | |
| EPI_ISL_4081550-EPI_ISL_4081551  (Ryt-Hansen et al. 2021) | 2016_17110_2p1/  2016 | 248-H1AN2D | N2sw | N2PA | N2PA | n/a | N2PA | N2PA | N2PA | N2PA | N2PA | |
| EPI_ISL_4081460-EPI_ISL_4081461  (Ryt-Hansen et al. 2021) | 2016_17747_3p2/  2016 | 250-H1AN1P | N1pdm09 | DSWP | DSWP | DSWP | DSWP | DSWP | DSWP | DSWP | DSWP | |
| PQ193335-PQ193342 | 16-17799-3/  2016 | 252-H1AN1P | N1pdm09 | DSWP | n/a | n/a | DSWP | DSWP | DSWP | n/a | n/a | |
| EPI_ISL_4081526-EPI_ISL_4081527  (Ryt-Hansen et al. 2021) | 2016_17837_1p1/  2016 | 253-H1AN1P | N1pdm09 | DSWP | DSWP | DSWP | DSWP | DSWP | DSWP | DSWP | DSWP | |
| EPI_ISL_4081446-EPI_ISL_4081447  (Ryt-Hansen et al. 2021) | 2016_18590_4p1/  2016 | 255-H1AN1P | N1pdm09 | DSWP | DSWP | DSWP | DSWP | DSWP | DSWP | DSWP | DSWP | |
| PQ193343-PQ193350 | 16-20178-1/  2016 | 257-H1AN2H | N2hu | N2HU | N2HU | N2HU | N2HU | N2HU | N2HU | N2HU | N2HU | |
| PQ193359-PQ193366 | 16-20978-1/  2016 | 259-H1AN1P | N1pdm09 | DSWP | DSWP | DSWP | DSWP | DSWP | DSWP | DSWP | DSWP | |
| EPI_ISL_304226  (Henritzi et al. 2020) | SIR147/  2017 | 415-H1AN1P | N1pdm09 | - | - | - | DSWP | - | DSWP | - | - | |
| PQ192850-PQ192856 | 17-281-3/  2017 | 289-H1AN1P | N1pdm09 | - | DSWP | DSWP | DSWP | DSWP | DSWP | DSWP | DSWP | |
| EPI_ISL_4081452-EPI_ISL_4081453  (Ryt-Hansen et al. 2021) | 2017_1287_4_4p1/  2017 | 279-H1AN1P | N1pdm09 | DSWP | DSWP | DSWP | DSWP | DSWP | DSWP | DSWP | DSWP | |
| EPI_ISL_4081564-EPI_ISL_4081565  (Ryt-Hansen et al. 2021) | 2017_2271_1_1p1/  2017 | 288-H1AN1P | N1pdm09 | HUMS | HUMS | HUMS | HUMS | HUMS | HUMS | HUMS | HUMS | |
| PQ193423-PQ193430 | 17-2911-1/  2017 | 291-H1AN1P | N1pdm09 | DSWP | DSWP | DSWP | DSWP | DSWP | DSWP | DSWP | DSWP | |
| PQ193431-PQ193438 | 17-2957-2/  2017 | 292-H1AN1P | N1pdm09 | DSWP | DSWP | DSWP | DSWP | DSWP | DSWP | DSWP | DSWP | |
| PQ193439-PQ193446 | 17-2989-1/  2017 | 293-H1AN1P | N1pdm09 | DSWP | DSWP | DSWP | DSWP | DSWP | DSWP | DSWP | DSWP | |
| EPI_ISL_4081454-EPI_ISL_4081455  (Ryt-Hansen et al. 2021) | 2017_3380_3_3p1/  2017 | 294-H1AN1P | N1pdm09 | DSWP | DSWP | DSWP | DSWP | DSWP | DSWP | DSWP | DSWP | |
| PQ193447-PQ193454 | 17-3482-2/  2017 | 296-H1AN2D | N2sw | DSWP | DSWP | DSWP | DSWP | n/a | n/a | DSWP | AV | |
| EPI_ISL_4081456-EPI_ISL_4081457  (Ryt-Hansen et al. 2021) | 2017_3423_1_1p1/  2017 | 295-H1AN1P | N1pdm09 | DSWP | DSWP | DSWP | DSWP | DSWP | DSWP | DSWP | DSWP | |
| PQ192857-PQ192863 | 17-4854-7/  2017 | 297-H1AN2H | N2hu | N2HU | - | N2HU | N2HU | - | N2HU | N2HU | N2HU | |
| PQ193455-PQ193462 | 17-5057-2/  2017 | 299-H1AN1P | N1pdm09 | n/a | n/a | n/a | DSWP | n/a | DSWP | n/a | n/a | |
| PQ193463-PQ193470 | 17-7735-1/  2017 | 301-H1AN2D | N2sw | HUMS | HUMS | HUMS | HUMS | n/a | n/a | HUMS | HUMS | |
| EPI_ISL_4081552-EPI_ISL_4081553  (Ryt-Hansen et al. 2021) | 2017_8009_3_3p1/  2017 | 303-H1AN2D | N2sw | N2PA | N2PA | n/a | N2PA | N2PA | N2PA | N2PA | N2PA | |
| PQ193471-PQ193478 | 17-9152-4/  2017 | 304-H1AN1P | N1pdm09 | n/a | n/a | n/a | DSWP | n/a | DSWP | n/a | n/a | |
| PQ193479-PQ193486 | 17-9838-2/  2017 | 305-H1AN1P | N1pdm09 | DSWP | DSWP | DSWP | DSWP | DSWP | DSWP | DSWP | DSWP | |
| PQ193487-PQ193494 | 17-9869-1/  2017 | 306-H1AN1P | N1pdm09 | DSWP | DSWP | DSWP | DSWP | DSWP | DSWP | DSWP | DSWP | |
| EPI_ISL_4081450-EPI_ISL_4081451  (Ryt-Hansen et al. 2021) | 2017_10298_4_4p1/  2017 | 272-H1AN1P | N1pdm09 | DSWP | DSWP | DSWP | DSWP | DSWP | DSWP | DSWP | DSWP | |
| EPI_ISL_4081538-EPI_ISL_4081539  (Ryt-Hansen et al. 2021) | 2017_11767_4_4p1/  2017 | 274-H1AN2D | N2sw | n/a | n/a | N2PA | n/a | n/a | n/a | n/a | n/a | |
| PQ193415-PQ193422 | 17-12364-4/  2017 | 277-H1AN1P | N1pdm09 | DSWP | DSWP | DSWP | DSWP | DSWP | DSWP | n/a | DSWP | |
| EPI_ISL_4081466-EPI_ISL_4081467  (Ryt-Hansen et al. 2021) | 2017_12409_3_3p1/  2017 | 278-H1AN1P | N1pdm09 | - | n/a | n/a | DSWP | n/a | DSWP | n/a | n/a | |
| EPI_ISL_4081492-EPI_ISL_4081508  (Ryt-Hansen et al. 2021) | 2017_15222_2_2p1/  2017 | 282-H1AN1P | N1pdm09 | DSWP | DSWP | DSWP | DSWP | DSWP | DSWP | DSWP | DSWP | |
| EPI_ISL_4081524-EPI_ISL_4081525  (Ryt-Hansen et al. 2021) | 2017_15824_1_1p1/  2017 | 283-H1AN1P | N1pdm09 | DSWP | DSWP | DSWP | DSWP | DSWP | DSWP | DSWP | DSWP | |
| EPI_ISL_4081406-EPI_ISL_4081407  (Ryt-Hansen et al. 2021) | 2018_57_3_1p1/  2018 | 339-H1AN1P | N1pdm09 | DSWP | DSWP | DSWP | DSWP | DSWP | DSWP | DSWP | DSWP | |
| EPI_ISL_4081462-EPI_ISL_4081463  (Ryt-Hansen et al. 2021) | 2018_2957_4_1p1/  2018 | 329-H1AN2D | N2sw | DSWP | DSWP | DSWP | DSWP | n/a | n/a | n/a | n/a | |
| PQ193567-PQ193574 | 18-2985-3/  2018 | 330-H1AN1V | N1av | N1AV | N1AV | N1AV | N1AV | N1AV | N1AV | N1AV | N1AV | |
| PQ192876-PQ192882 | 18-4510-2/  2018 | 333-H1AN1P | N1pdm09 | - | DSWP | DSWP | DSWP | DSWP | DSWP | DSWP | DSWP | |
| PQ193575-PQ193582 | 18-5071-2/  2018 | 336-H1AN1P | N1pdm09 | DSWP | DSWP | DSWP | DSWP | DSWP | DSWP | DSWP | DSWP | |
| PQ193583-PQ193590 | 18-5109-1/  2018 | 337-H1AN1P | N1pdm09 | DSWP | DSWP | DSWP | DSWP | DSWP | DSWP | DSWP | DSWP | |
| PQ193591-PQ193598 | 18-5269-3/  2018 | 338-H1AN1P | N1pdm09 | DSWP | DSWP | DSWP | DSWP | DSWP | DSWP | DSWP | DSWP | |
| PQ193599-PQ193606 | 18-6936-3/  2018 | 340-H1AN1P | N1pdm09 | DSWP | DSWP | DSWP | DSWP | DSWP | DSWP | DSWP | DSWP | |
| PQ193607-PQ193614 | 18-9602-3/  2018 | 341-H1AN1P | N1pdm09 | n/a | n/a | n/a | DSWP | n/a | DSWP | n/a | n/a | |
| PQ193495-PQ193502 | 18-10004-2/  2018 | 307-H1AN1P | N1pdm09 | DSWP | DSWP | DSWP | DSWP | DSWP | DSWP | DSWP | DSWP | |
| PQ193503-PQ193510 | 18-10009-2/  2018 | 308-H1AN1P | N1pdm09 | DSWP | DSWP | DSWP | DSWP | DSWP | DSWP | DSWP | DSWP | |
| PQ193511-PQ193518 | 18-11095-1/  2018 | 309-H1AN2D | N2sw | DSWP | DSWP | DSWP | DSWP | DSWP | n/a | n/a | DSWP | |
| EPI_ISL_4081420-EPI_ISL_4081435  (Ryt-Hansen et al. 2021) | 2018_11980_2_1p1/  2018 | 313-H1AN2D | N2sw | n/a | n/a | n/a | DSWP | n/a | n/a | DSWP | n/a | |
| PQ193519-PQ193526 | 18-11212-1/  2018 | 310-H1AN1P | N1pdm09 | DSWP | DSWP | DSWP | DSWP | DSWP | DSWP | DSWP | DSWP | |
| EPI_ISL_4081444-EPI_ISL_4081445  (Ryt-Hansen et al. 2021) | 2018_11784_2_1p1/  2018 | 311-H1AN1P | N1pdm09 | DSWP | DSWP | DSWP | DSWP | DSWP | DSWP | DSWP | DSWP | |
| EPI_ISL_4081468-EPI_ISL_4081469  (Ryt-Hansen et al. 2021) | 2018_12354_2_1p1/  2018 | 315-H1AN1P | N1pdm09 | n/a | n/a | n/a | DSWP | n/a | DSWP | n/a | n/a | |
| PQ193527-PQ193534 | 18-11933-1/  2018 | 312-H1AN1P | N1pdm09 | DSWP | DSWP | DSWP | DSWP | DSWP | n/a | DSWP | DSWP | |
| PQ193535-PQ193542 | 18-13154-2/  2018 | 316-H1AN1P | N1pdm09 | DSWP | DSWP | DSWP | DSWP | DSWP | DSWP | DSWP | DSWP | |
| PQ192864-PQ192870 | 18-13175-2/  2018 | 317-H1AN2D | N2sw | N2PA | N2PA | N2PA | N2PA | N2PA | N2PA | N2PA | - | |
| EPI_ISL_4081408-EPI_ISL_4081409  (Ryt-Hansen et al. 2021) | 2018_13984_3_1p1/  2018 | 318-H1AN2D | N2sw | DSWP | DSWP | DSWP | DSWP | DSWP | n/a | n/a | DSWP | |
| PQ193543-PQ193550 | 18-14628-1/  2018 | 320-H1AN1P | N1pdm09 | DSWP | DSWP | DSWP | DSWP | DSWP | DSWP | DSWP | DSWP | |
| EPI_ISL_4081402-EPI_ISL_4081403  (Ryt-Hansen et al. 2021) | 2018_15183_3_1p1/  2018 | 322-H1AN2D | N2sw | n/a | n/a | N2PA | DSWP | n/a | n/a | n/a | n/a | |
| PQ193551-PQ193558 | 18-14957-2/  2018 | 321-H1AN2D | N2sw | DSWP | DSWP | DSWP | DSWP | DSWP | n/a | n/a | DSWP | |
| PQ193559-PQ193566 | 18-15403-3/  2018 | 323-H1AN1P | N1pdm09 | DSWP | DSWP | DSWP | DSWP | DSWP | DSWP | DSWP | DSWP | |
| PQ192871-PQ192875 | 18-15808-4/  2018 | 324-H1AN1P | N1pdm09 | - | - | - | DSWP | DSWP | DSWP | DSWP | DSWP | |
| PQ193655-PQ193662 | 19-155-3/  2019 | 356-H1AN1P | N1pdm09 | DSWP | DSWP | DSWP | DSWP | DSWP | DSWP | DSWP | DSWP | |
| PQ192890-PQ192896 | 19-361-1/  2019 | 359-H1AN1P | N1pdm09 | - | DSWP | DSWP | DSWP | DSWP | DSWP | DSWP | DSWP | |
| PQ115440-PQ115447 | 19-1556-4/  2019 | 357-H1AN2D | N2sw | n/a | n/a | n/a | n/a | n/a | n/a | n/a | n/a | |
| PQ115401-PQ115408 | 19-5090-1/  2019 | 370-H1AN1P | N1pdm09 | DSWP | n/a | DSWP | DSWP | n/a | DSWP | n/a | DSWP | |
| PQ115666-PQ115673 | 19-50292-1/  2019 | 364-H1AN1P | N1pdm09 | HUMS | HUMS | HUMS | HUMS | HUMS | HUMS | HUMS | - | |
| PQ115409-PQ115416 | 19-50299-5/  2019 | 365-H1AN1P | N1pdm09 | DSWP | DSWP | DSWP | DSWP | DSWP | DSWP | DSWP | DSWP | |
| PQ115674-PQ115681 | 19-50449-4/  2019 | 366-H1AN1P | N1pdm09 | n/a | n/a | n/a | DSWP | n/a | DSWP | n/a | n/a | |
| PQ115417-PQ115423 | 19-50470-5/  2019 | 368-H1AN1P | N1pdm09 | n/a | - | HUMS | HUMS | n/a | HUMS | n/a | - | |
| PQ193639-PQ193646 | 19-10657-1/  2019 | 353-H1AN1V | N1av | N1AV | N1AV | N1AV | N1AV | N1AV | N1AV | N1AV | N1AV | |
| PQ193647-PQ193654 | 19-12219-2/  2019 | 354-H1AN1V | N1av | N1AV | N1AV | N1AV | N1AV | N1AV | N1AV | N1AV | N1AV | |
| PQ115695 | 19-S01822-2/  2019 | 343-H1AN2D | N2sw | - | - | - | - | - | n/a | - | - | |
| PQ115714-PQ115721 | 19-02059-2/  2019 | 346-H1AN1V | N1av | N1AV | N1AV | N1AV | N1AV | N1AV | N1AV | N1AV | N1AV | |
| PQ115697-PQ115705 | 19-01969-3/  2019 | 344-H1AN1V | N1av | N1AV | N1AV | N1AV | N1AV | N1AV | N1AV | N1AV | N1AV | |
| PQ115432-PQ115439 | 19-S02060-1/  2019 | 347-H1AN1P | N1pdm09 | - | - | - | n/a | - | n/a | - | AV | |
| PQ115424-PQ115431 | 19-02109-3/  2019 | 349-H1AN1P | N1pdm09 | n/a | n/a | n/a | DSWP | n/a | DSWP | n/a | n/a | |
| PQ115634-PQ115641 | 19-02142-2/  2019 | 351-H1AN1V | N1av | N1AV | N1AV | N1AV | N1AV | N1AV | N1AV | N1AV | N1AV | |
| PQ115448-PQ115454 | 19-S02108-3/  2019 | 348-H1AN1V | N1av | N1AV | N1AV | N1AV | N1AV | N1AV | N1AV | N1AV | N1AV | |
| PQ192883-PQ192889 | 19-14890-3/  2019 | 355-H1AN2D | N2sw | DSWP | DSWP | DSWP | DSWP | DSWP | n/a | n/a | DSWP | |
| PQ193679-PQ193686 | 20-2444-1/  2020 | 372-H1AN1P | N1pdm09 | DSWP | n/a | DSWP | DSWP | DSWP | DSWP | n/a | AV | |
| PQ193695-PQ193702 | 20-3115-5/  2020 | 374-H1AN2D | N2sw | n/a | n/a | N2PA | n/a | n/a | n/a | n/a | n/a | |
| PQ193687-PQ193694 | 20-3114-3/  2020 | 373-H1AN1P | N1pdm09 | HUMS | HUMS | HUMS | HUMS | HUMS | HUMS | HUMS | HUMS | |
| PQ193703-PQ193710 | 20-3199-2/  2020 | 375-H1AN1P | N1pdm09 | HUMS | HUMS | n/a | HUMS | n/a | HUMS | HUMS | HUMS | |
| PQ115914-PQ115921 | 20-S03246-1/  2020 | 376-H1AN1V | N1av | N1AV | N1AV | N1AV | N1AV | N1AV | N1AV | N1AV | N1AV | |
| PQ115810-PQ115817 | 20-3343-3/  2020 | 377-H1AN1V | N1av | N1AV | N1AV | N1AV | N1AV | N1AV | N1AV | N1AV | N1AV | |
| PQ115818-PQ115825 | 20-3710-1/  2020 | 378-H1AN1V | N1av | N1AV | N1AV | N1AV | N1AV | N1AV | N1AV | N1AV | N1AV | |
| PQ115826-PQ115833 (Nissen et al. 2021) | 20-3711-1/  2020 | 379-H1AN1V | N1av | N1AV | N1AV | N1AV | N1AV | N1AV | N1AV | N1AV | N1AV | |
| PQ193743-PQ193750 (Nissen et al. 2021) | 20-3797-4/  2020 | 380-H1AN1P | N1pdm09 | DSWP | DSWP | DSWP | DSWP | DSWP | DSWP | DSWP | AV | |
| PQ193751-PQ193758 | 20-3851-3/  2020 | 381-H1AN1P | N1pdm09 | DSWP | DSWP | DSWP | DSWP | DSWP | DSWP | DSWP | AV | |
| PQ193759-PQ193766 | 20-3884-2/  2020 | 382-H1AN1V | N1av | N1AV | N1AV | N1AV | N1AV | N1AV | N1AV | N1AV | N1AV | |
| PQ193767-PQ193774 | 20-3914-2/  2020 | 383-H1AN1P | N1pdm09 | DSWP | DSWP | DSWP | DSWP | DSWP | n/a | DSWP | DSWP | |
| PQ115834-PQ115841 | 20-3955-1/  2020 | 384-H1AN1V | N1av | N1AV | N1AV | N1AV | N1AV | N1AV | N1AV | N1AV | N1AV | |
| PQ193783-PQ193790 | 20-4018-5/  2020 | 385-H1AN1P | N1pdm09 | n/a | n/a | n/a | HUMS | n/a | HUMS | n/a | n/a | |
| PQ115527-PQ115534 | 20-4071-5/  2020 | 386-H1AN1V | N1av | N1AV | N1AV | N1AV | N1AV | N1AV | N1AV | N1AV | N1AV | |
| PQ115842-PQ115849 | 20-4127-2/  2020 | 387-H1AN1V | N1av | N1AV | N1AV | N1AV | N1AV | N1AV | N1AV | N1AV | N1AV | |
| PQ193799-PQ193806 (Andersen et al. 2022) | 20-4291-1/  2020 | 388-H1AN2D | N2sw | n/a | N1AV | n/a | N1AV | n/a | n/a | N1AV | N1AV | |
| PQ115930-PQ115937 | 20-S04292-4/  2020 | 389-H1AN1P | N1pdm09 | n/a | n/a | n/a | DSWP | n/a | DSWP | n/a | n/a | |
| PQ115850-PQ115857 | 20-4761-5/  2020 | 390-H1AN1V | N1av | DSWP | N1AV | DSWP | N1AV | N1AV | N1AV | N1AV | N1AV | |
| PQ115858-PQ115865 | 20-4762-3/  2020 | 391-H1AN1V | N1av | N1AV | N1AV | N1AV | N1AV | N1AV | N1AV | N1AV | N1AV | |
| PQ193823-PQ193830 | 20-4762-5/  2020 | 392-H1AN1V | N1av | N1AV | N1AV | N1AV | N1AV | N1AV | N1AV | N1AV | N1AV | |
| PQ115866-PQ115873 | 20-4767-4/  2020 | 393-H1AN1V | N1av | N1AV | N1AV | N1AV | N1AV | N1AV | N1AV | N1AV | N1AV | |
| PQ115938-PQ115945 | 20-S05032-3/  2020 | 394-H1AN1V | N1av | N1AV | N1AV | N1AV | N1AV | N1AV | N1AV | N1AV | N1AV | |
| PQ115954-PQ115961 | 20-S05482-2/  2020 | 395-H1AN1P | N1pdm09 | DSWP | DSWP | DSWP | DSWP | DSWP | DSWP | DSWP | DSWP | |
| PQ192904-PQ192910 | 20-5522-3/  2020 | 396-H1AN1P | N1pdm09 | - | DSWP | DSWP | DSWP | DSWP | n/a | DSWP | DSWP | |
| PQ115962-PQ115969 | 20-S06030-4/  2020 | 397-H1AN2D | N2sw | DSWP | DSWP | DSWP | DSWP | DSWP | n/a | n/a | DSWP | |
| PQ192911-PQ192918 | 20-6454-1/  2020 | 398-H1AN1P | N1pdm09 | n/a | n/a | n/a | DSWP | n/a | DSWP | n/a | n/a | |
| PQ115874-PQ115881 | 20-6872-2/  2020 | 399-H1AN1V | N1av | N1AV | N1AV | N1AV | N1AV | N1AV | N1AV | N1AV | N1AV | |
| PQ193871-PQ193878 | 20-8035-3/  2020 | 400-H1AN1P | N1pdm09 | DSWP | DSWP | DSWP | DSWP | DSWP | n/a | n/a | DSWP | |
| PQ115882-PQ115889 | 20-8233-2/  2020 | 401-H1AN1V | N1av | N1AV | N1AV | N1AV | N1AV | N1AV | N1AV | N1AV | N1AV | |
| PQ115890-PQ115897 | 20-8396-1/  2020 | 402-H1AN1V | N1av | N2PA | N1AV | N2PA | N1AV | N2PA | N1AV | N1AV | N1AV | |
| PQ115898-PQ115905 | 20-8654-4/  2020 | 403-H1AN1V | N1av | N1AV | N1AV | N1AV | N1AV | N1AV | N1AV | N1AV | N1AV | |
| PQ115498-PQ115505 | 20-S09755-2/  2020 | 404-H1AN1P | N1pdm09 | n/a | HUMS | n/a | HUMS | DSWP | HUMS | n/a | n/a | |
| PQ115906-PQ115913 | 20-S010445-1/  2020 | 371-H1AN1P | N1pdm09 | DSWP | DSWP | DSWP | DSWP | DSWP | n/a | n/a | DSWP | |
| EPI_ISL_9604273 (Andersen et al. 2022) | S19922-5/  2021 | 410-H1AN1V | N1av | DSWP | n/a | DSWP | N1AV | DSWP | N1AV | n/a | AV | |
| EPI_ISL_9605757 (Andersen et al. 2022) | S23212-3/  2021 | 412-H1AN1V | N1av | - | n/a | - | N1AV | DSWP | N1AV | n/a | AV | |
| EPI_ISL_9606508 (Andersen et al. 2022) | S23620-3/  2021 | 413-H1AN1V | N1av | - | - | - | N1AV | DSWP | N1AV | - | AV | |
| EPI_ISL_9606987 (Andersen et al. 2022) | S24795-1/  2021 | 414-H1AN2D | N2sw | - | n/a | DSWP | N1AV | DSWP | n/a | n/a | AV | |
| EPI_ISL_909652  (Nissen et al. 2021) | *A/Denmark/1/2021 | 20-H1AN1P | N1pdm09 | DSWP | DSWP | DSWP | DSWP | DSWP | DSWP | DSWP | AV | |
| EPI_ISL_8786194 (Andersen et al. 2022) | *A/Denmark/36/2021 | 24-H1AN1V | N1av | DSWP | n/a | DSWP | N1AV | DSWP | N1AV | n/a | AV | |

**Supplementary Table 2)** Rates of evolution (number of nucleotide substitutions per site per year) averaged across each genomic segment phylogeny with one standard deviation (+/-). Numbers of sequences in each phylogenetic tree and nucleotide lengths of each genomic segment coding region are indicated. r^2^ measured the goodness-of-fit for the root-to-tip regression model on a 0-1 scale, and higher chi^2^ scores were indicative of greater differences between the observed and expected values in the model.

| **Segment phylogeny (expressed proteins); lineage** | **Sequences** | **Nucleotides per genomic segment** | **Nucleotide substitutions per site per year** | **chi^2^** | **r^2^** |
| --- | --- | --- | --- | --- | --- |
| PB2; pdm09 | 377 | 2280 | 2.92 x 10^-3^ +/- 7.32 x 10^-5^ | 15358 | 0.87 |
| PB1; pdm09 | 379 | 2274 | 2.97 x 10^-3^ +/- 7.31 x 10^-5^ | 14940 | 0.90 |
| PA (PA/PA-X); pdm09 | 380 | 2151 | 2.82 x 10^-3^ +/- 7.29 x 10^-5^ | 7163 | 0.93 |
| HA; pdm09 | 390 | 1701 | 4.58 x 10^-3^ +/- 1.10 x 10^-4^ | 21344 | 0.83 |
| NP; pdm09 | 397 | 1497 | 2.72 x 10^-3^ +/- 9.57 x 10^-5^ | 6908 | 0.86 |
| NA; pdm09 | 303 | 1410 | 3.66 x 10^-3^ +/- 1.40 x 10^-4^ | 4787 | 0.92 |
| NA; N1av | 53 | 1410 | 3.14 x 10^-3^ +/- 1.55 x 10^-4^ | 217 | 0.95 |
| NA; N2sw | 126 | 1410 | 3.70 x 10^-3^ +/- 1.28 x 10^-4^ | 2975 | 0.86 |
| NA; N2hu | 24 | 1410-1422 | 3.64 x 10^-3^ +/- 1.62 x 10^-4^ | 52 | 0.96 |
| M (M1/M2); pdm09 | 393 | 982 | 1.97 x 10^-3^ +/- 1.11 x 10^-4^ | 2567 | 0.79 |
| NS (NS1/NEP); pdm09 | 371 | 838 | 2.73 x 10^-3^ +/- 1.44 x 10^-4^ | 2209 | 0.85 |
| NS (NS1/NEP); av | 56 | 838 | 2.50 x 10^-3^ +/- 1.60 x 10^-4^ | 736 | 0.90 |

**Supplementary Table 3)** Positive selection acted on the formation of clades DSWP and N1AV of clade-forming branches in HA segment phylogeny as detected by aBSREL. Ratio of non-synonymous to synonymous evolutionary rates (ω) were divided into two selection rate classes, ω_1_ (neutral) and ω_2_ (positive), and distributed over proportions of codon sites. Statistical significance was evaluated by likelihood ratio test set at test p-value ≤ 0.05 following multiple test correction. Amino acid substitutions (H1 numbering) inherited along the branches are listed and sites of mutations inherited by both branches are underlined.

| Clade-forming branch | Likelihood ratio test | Test p-value | Uncorrected p-value | ω distribution over sites | Amino acid substitutions |
| --- | --- | --- | --- | --- | --- |
| DSWP | 12.1457 | 0.0142 | 0.0008 | ω_1_ = 0.242 (100%)  ω_2_ = 149 (0.22%) | N16D, H143N, D144N, N146K, K147E, A152S, K159S, V172T, S179N, K180I, T202A, S207R, D291E, V338D |
| N1AV | 12.2228 | 0.0144 | 0.0008 | ω_1_ = 0.932 (99%)  ω_2_ = 1050 (0.55%) | L87I, T99A, Q121H, T137A, K159N, V169I, G172E, K180I, I193V, T207W, N211D, D213N, R222K, V267A, T287A, V289I, K325R, K328R, I338T, R467K, S468G, R526K |

**Supplementary Table 4)** Selection pressures acted on the evolution of individual codon sites as detected by three site-level selection models, MEME, FEL and SLAC. Number of sequences in each phylogeny after removal of identical sequences and the maximum number of codon sites in each protein are listed. Number of codon sites under positive or negative selection are provided as a proportion of the encoded protein with significant p-values set at ≤0.1. Ratio of non-synonymous to synonymous evolutionary rate (ω) was estimated for the whole gene by MEME and SLAC.

| Protein (origin) | Sequences | Codons | Positive/diversifying selection (% of codons in protein) | | | Negative/purfy-ing selection (% of codons in protein) | | ω | |
| --- | --- | --- | --- | --- | --- | --- | --- | --- | --- |
|  |  |  | MEME | FEL | SLAC | FEL | SLAC | MEME | SLAC |
| PB2 | 365 | 759 | 0.92 | 0.66 | 0.53 | 68.25 | 61.92 | 0.09 | 0.10 |
| PB1 | 367 | 757 | 0.92 | 0.40 | 0.26 | 70.94 | 63.54 | 0.09 | 0.10 |
| PA | 373 | 716 | 1.26 | 0.70 | 0.84 | 68.72 | 62.43 | 0.10 | 0.11 |
| PA-X | 308 | 253 | 7.11 | 7.51 | 5.14 | 52.17 | 48.22 | 0.19 | 0.21 |
| HA | 380 | 566 | 4.06 | 3.53 | 3.18 | 58.30 | 51.59 | 0.27 | 0.29 |
| NP | 362 | 498 | 1.00 | 0.60 | 0.80 | 72.29 | 64.26 | 0.07 | 0.08 |
| NA (N1pdm09) | 294 | 469 | 2.56 | 1.49 | 1.92 | 50.53 | 40.51 | 0.22 | 0.26 |
| NA (N2sw) | 123 | 469 | 2.77 | 1.49 | 1.28 | 64.61 | 56.93 | 0.18 | 0.21 |
| NA (N1av) | 52 | 469 | 1.49 | 2.13 | 0.43 | 46.70 | 36.67 | 0.18 | 0.22 |
| NA (N2hu) | 24 | 469 | 0.43 | 0.43 | 0.43 | 36.67 | 15.57 | 0.18 | 0.21 |
| M1 | 308 | 252 | 1.19 | 0.40 | 0.40 | 66.27 | 53.97 | 0.05 | 0.05 |
| M2 | 171 | 97 | 8.25 | 6.19 | 5.15 | 23.71 | 14.43 | 0.55 | 0.58 |
| NS1 (pdm09) | 306 | 230 | 6.96 | 6.09 | 3.48 | 37.83 | 31.74 | 0.35 | 0.36 |
| NS1 (av) | 55 | 230 | 3.04 | 2.17 | 0.87 | 28.26 | 17.39 | 0.31 | 0.33 |
| NEP (pdm09) | 223 | 121 | 2.48 | 1.65 | 0.00 | 38.84 | 30.58 | 0.26 | 0.28 |
| NEP (av) | 53 | 121 | 0.83 | 0.83 | 0.00 | 27.27 | 15.70 | 0.21 | 0.23 |

**Supplementary Table 5)** List of amino acid (codon) sites evolving under positive selection across the phylogenies (H1 numbering used for HA codon sites). Grey scale shading corresponds to the site-based models used for selection detection; dark grey detected by MEME model only, light grey detected by MEME and FEL and/or SLAC, and white codons detected by FEL and/or SLAC models only.

| PB2 | PB1 | PA | PA-X | HA | NP | N1 | N1 | M1 | M2 | NS1 | NS1 av | NEP pdm09 |
| --- | --- | --- | --- | --- | --- | --- | --- | --- | --- | --- | --- | --- |
|  |  |  |  |  |  | pdm09 | av |  |  | pdm09 |  |  |
| 106 | 108 | 70 | 199 | 3 | 34 | 23 | 5 | 30 | 10 | 56 | 108 | 26 |
| 127 | 435 | 213 | 204 | 16 | 53 | 34 | 12 | 214 | 13 | 60 | 113 | 32 |
| 184 | 527 | 321 | 206 | 62 | 101 | 74 | 59 |  | 14 | 76 | 127 | 33 |
| 456 | 584 | 330 | 208 | 65 | 217 | 75 | 75 |  | 20 | 147 | 180 |  |
| 478 | 644 | 362 | 209 | 86 | 473 | 106 | 78 |  | 21 | 153 | 194 |  |
| 559 | 737 | 387 | 210 | 90 |  | 200 | 200 |  | 23 | 171 | 210 |  |
|  | 757 | 396 | 212 | 99 |  | 232 | 343 |  | 27 | 178 | 213 |  |
|  |  | 400 | 213 | 100 |  | 240 | 396 |  | 30 | 180 | 227 |  |
|  |  | 543 | 216 | 137 |  | 248 | 454 |  |  | 182 |  |  |
|  |  | 614 | 218 | 142 |  | 249 | 466 |  |  | 189 |  |  |
|  |  |  | 221 | 145 |  | 257 |  |  |  | 192 |  |  |
|  |  |  | 222 | 149 |  | 270 |  |  |  | 205 |  |  |
|  |  |  | 226 | 158 |  | 334 |  |  |  | 208 |  |  |
|  |  |  | 228 | 159 |  | 394 |  |  |  | 209 |  |  |
|  |  |  | 229 | 174 |  | 432 |  |  |  | 212 |  |  |
|  |  |  |  | 179 |  |  |  |  |  | 215 |  |  |
|  |  |  |  | 183 |  |  |  |  |  |  |  |  |
|  |  |  |  | 200 |  |  |  |  |  |  |  |  |
|  |  |  |  | 202 |  |  |  |  |  |  |  |  |
|  |  |  |  | 203 |  |  |  |  |  |  |  |  |
|  |  |  |  | 204 |  |  |  |  |  |  |  |  |
|  |  |  |  | 206 |  |  |  |  |  |  |  |  |
|  |  |  |  | 207 |  |  |  |  |  |  |  |  |
|  |  |  |  | 277 |  |  |  |  |  |  |  |  |
|  |  |  |  | 387 |  |  |  |  |  |  |  |  |
|  |  |  |  | 544 |  |  |  |  |  |  |  |  |
|  |  |  |  | 550 |  |  |  |  |  |  |  |  |

**Supplementary Table 6**) Relative prevalence of predicted N-linked glycosylation on HA protein sites (H1 numbering) in H1N1 pandemic 2009 (H1N1pdm09)-related populations. Only sites with a relative prevalence above 25 % in at least one defined population are displayed. Viral populations are defined as human (early pandemic (2009-2012) or seasonal (2013-2020), zoonotic influenza A viruses (IAVs), and Danish swine IAV (swIAV) H1pdm09Nx separated by NA subtype and reverse zoonotic swIAVs (REVZ). Glycan sites on zoonotic IAVs, A/Denmark/1/2021 (DK01) and A/Denmark/36/2021 (DK36), are indicated as present (🗸) or absent (-).

| HA protein site | Relative prevalence (%) in H1pdm09Nx populations | | | | | | | | |
| --- | --- | --- | --- | --- | --- | --- | --- | --- | --- |
|  | Human | | Danish swIAV (2010-2020) | | | | | Zoonotic | |
|  | Early (2009-2012) | Seasonal (2013-2020) | REVZ | N1 pdm09 | N1av | N2sw | N2hu | DK01 | DK36 |
| 28 | 98 | 100 | 100 | 100 | 100 | 100 | 100 | **🗸** | **🗸** |
| 40 | 98 | 100 | 100 | 100 | 97 | 100 | 100 | **🗸** | **🗸** |
| 104 | 100 | 100 | 100 | 99 | 100 | 100 | 100 | **🗸** | **🗸** |
| 136 | 2 | 0 | 0 | 10 | 97 | 67 | 38 | - | **🗸** |
| 179 | 6 | 41 | 67 | 56 | 10 | 17 | 25 | **🗸** | **🗸** |
| 202 | 0 | 0 | 0 | 3 | 94 | 61 | 0 | - | - |
| 293 | 0 | 0 | 0 | 48 | 100 | 22 | 0 | **🗸** | **🗸** |
| 304 | 98 | 100 | 100 | 99 | 74 | 100 | 100 | **🗸** | **🗸** |
| 498 | 96 | 98 | 100 | 71 | 100 | 100 | 25 | **🗸** | **🗸** |
